# Annotation, classification, genomic organization and expression of the *Vitis vinifera* CYPome

**DOI:** 10.1371/journal.pone.0199902

**Published:** 2018-06-28

**Authors:** Tina Ilc, Gautier Arista, Raquel Tavares, Nicolas Navrot, Eric Duchêne, Amandine Velt, Frédéric Choulet, Etienne Paux, Marc Fischer, David R. Nelson, Philippe Hugueney, Danièle Werck-Reichhart, Camille Rustenholz

**Affiliations:** 1 Institute of Plant Molecular Biology, Centre National de la Recherche Scientifique, Université de Strasbourg, Strasbourg, France; 2 Université de Strasbourg, INRA, SVQV UMR-A 1131, Colmar, France; 3 Laboratoire de Biométrie et Biologie Evolutive, Centre National de la Recherche Scientifique, Université de Lyon 1, Lyon, France; 4 Laboratoire Structure et Evolution du Génome du Blé, Institut National de la Recherche Agronomique, Université Blaise Pascal, Clermont-Ferrand, France; 5 Department of Microbiology, Immunology and Biochemistry, University of Tennessee Health Science Center, Memphis, Tennessee, United States of America; Universidade de Lisboa Instituto Superior de Agronomia, PORTUGAL

## Abstract

Cytochromes P450 are enzymes that participate in a wide range of functions in plants, from hormonal signaling and biosynthesis of structural polymers, to defense or communication with other organisms. They represent one of the largest gene/protein families in the plant kingdom. The manual annotation of cytochrome P450 genes in the genome of *Vitis vinifera* PN40024 revealed 579 P450 sequences, including 279 complete genes. Most of the P450 sequences in grapevine genome are organized in physical clusters, resulting from tandem or segmental duplications. Although most of these clusters are small (2 to 35, median = 3), some P450 families, such as CYP76 and CYP82, underwent multiple duplications and form large clusters of homologous sequences. Analysis of gene expression revealed highly specific expression patterns, which are often the same within the genes in large physical clusters. Some of these genes are induced upon biotic stress, which points to their role in plant defense, whereas others are specifically activated during grape berry ripening and might be responsible for the production of berry-specific metabolites, such as aroma compounds. Our work provides an exhaustive and robust annotation including clear identification, structural organization, evolutionary dynamics and expression patterns for the grapevine cytochrome P450 families, paving the way to efficient functional characterization of genes involved in grapevine defense pathways and aroma biosynthesis.

## Introduction

Grapevine (*Vitis vinifera L*.) is one of the oldest [1] and economically the most important [2] fruit crop in the world. The majority of grapes produced worldwide are used in winemaking. Modern cultivated grapevine has been shaped by thousands of years of selection for traits such as berry size, sugar content or skin color [3], but today’s viticulture is facing new challenges. In addition to pathogen pressure, it has to deal with climate change [4,5], and shift of consumer preference towards higher quality wines with a lower environmental impact [6,7]. Traditional breeding is extremely difficult to apply in grapevine because of its long lifecycle, reduced fitness of progeny and complexity of quality traits [8]. Sequencing of the grapevine genome in 2007 [9] and advances in the ‘omics’ techniques [10] set the stage for more efficient breeding solutions. The next crucial step towards improved grapevine varieties is the identification of genes underlying important traits, such as response towards pathogens, fruit development and quality.

Many developmental as well as ecological response pathways in plants involve cytochrome P450 oxygenases [11,12]. In plants, these enzymes catalyze regio- and stereospecific insertion of an oxygen atom into small, hydrophobic substrates that range from terpenoids and fatty acids to amino acids and their derivatives, such as phenolic compounds. In the model plant *Arabidopsis thaliana* they control processes as diverse as plant growth and branching [13,14], flower [15,16] and fruit development [17], formation of lignin and surface biopolymers [18,19], emission of volatiles [20,21] or plant-pathogen and plant-insect interactions [21–23]. In crop plants, P450s play major roles in shaping agriculturally-relevant traits, such as fruit size [24] or aroma biosynthesis [25]. This makes cytochromes P450 attractive targets for crop improvement.

Cytochromes P450 in plants evolved into many distinct families, which are usually composed by genes with 40% or higher protein sequence identity. Within one P450 family the biochemical function is often conserved across the plant kingdom. For example, enzymes from the CYP97 family are involved in carotenoid hydroxylation, CYP79s in the *N*-hydroxylation of amino acid to aldoximes, CYP75s in the hydroxylation of flavonoids, and CYP704s in addition to CYP703s in fatty acid hydroxylation to form the precursors to structural polymers sporopollenin and cutin [26]. Members of other families, however, have divergent functions: some members of CYP72 family are involved in iridoid biosynthesis, whereas others oxidize triterpene substrates [27]. These differences stem from different evolutionary pressures on genes with different functions. Families with essential functions, such as hormone metabolism or synthesis of biopolymers, usually show a low copy number and are submitted to high purifying selection, whereas families with adaptive functions expanded or “bloomed” in certain taxa [28]. A well-documented example is the bloom of the CYP76M subfamily in rice (*Oryza sativa*), which consists of 11 genes and 2 pseudogenes. At least 4 members of this subfamily are involved in the biosynthesis of diterpenoid antifungal compounds [29,30]. They are clustered close together in the genome, which is another common feature of recently duplicated P450s and probably result from sequential tandem duplications [28]. Interestingly, in other plants, for example *Arabidopsis thaliana* or *Catharanthus roseus*, some CYP76 members have a different biochemical function, namely oxidation of monoterpenols or their iridoid derivatives [31,32]. Recently expanded P450 families might therefore have new ecological functions, but those are more difficult to predict compared to functions of conserved P450 families. In addition, function of many P450 families is still unknown or poorly understood.

A previous annotation of P450s has highlighted some potentially interesting gene families in the highly heterozygous *V*. *vinifera* cv. Pinot Noir genome [33–35]. In this work we performed the first complete manual annotation of P450s in the nearly homozygous *V*. *vinifera* reference genome PN40024 [9]. We discuss the structural organization of the genes with particular focus on gene clusters. We evaluate phylogenetic relationships between those genes in order to be able to identify recently expanded gene families likely linked to adaptive traits or domestication. Finally, we investigate spatio-temporal gene expression patterns, with particular focus on berry development and pathogen response to detect P450s with potential roles in these important physiological processes.

## Material and methods

### Gene annotation

We annotated the cytochromes P450 using the 12X.2 version of the assembly of the *Vitis vinifera* cv PN40024 genome ([9,36], https://urgi.versailles.inra.fr/Species/Vitis/Data-Sequences/Genome-sequences). Four publically available datasets of cytochromes P450 were used to perform similarity searches in the PN40024 genome. 947 protein sequences of grape P450s were downloaded from the NCBI Protein database (http://www.ncbi.nlm.nih.gov/protein, Feb 2014). Three datasets were downloaded from David Nelson’s website (http://drnelson.uthsc.edu/CytochromeP450.html, Feb 2014), which stores manually curated annotations of cytochromes P450 for many species: 702 P450 protein sequences of *Vitis vinifera* cv Pinot Noir clone ENTAV115 (28, http://drnelson.uthsc.edu/vitis.htm); 416 P450 protein sequences of *Vitis vinifera* cv PN40024 from the 8x assembly version of the genome (10, http://drnelson.uthsc.edu/Vitis.additionalP450s.htm); and 288 P450 protein sequences of *Arabidopsis thaliana* (35, http://drnelson.uthsc.edu/Arabidopsis.Blast.file.html). Using these four datasets, we expected to be as exhaustive as possible in the cytochromes P450 similarly search of the PN40024 genome. The four datasets were masked for repeat sequences using the online tool “Repeat Masking” from Censor (http://www.girinst.org/censor/index.php).

The four masked datasets were used to perform four independent TBLASTN analyses [37] against the PN40024 12X.2 sequence with an e-value cutoff of 1e-3. The TBLASTN outputs were parsed using a homemade script. The hits from the three grape datasets were kept if they were at least 50 amino acids long with at least 70% sequence identity. The hits from the *Arabidopsis* dataset were kept if they were at least 50 amino acids long with an identity percentage of at least 50%. The software Exonerate (version 2.2.0, build October 2008, [38]) was used to predict gene structures using the protein2genome parameter and the same cutoff of sequence identity as above. A homemade script was used to reformat the output files from exonerate into files in the gff format. These gff files were imported to the Artemis genome browser [39] to perform the manual curation of the structures suggested by Exonerate. The parsed hits identified through TBLASTN were used to improve or to complete the Exonerate annotations. Every annotation starting with a start codon, ending with a stop codon and with correct exon-intron borders (GT-AG or sometimes GC-AG) was considered as a complete “gene”. Every annotation showing this gene structure (start and stop codons, correct exon-intron borders) but with a single point mutation creating a frameshift, a premature stop codon or a wrong exon-intron border was considered as a “putative pseudogene” also marked “pseudogene?” because it may result from a mistake in the genome assembly. Every annotation interrupted by a gap in the genomic sequence or including one was considered as a “partial” annotation. All the other annotations with wrong gene structure but showing a significant similarity level with a cytochrome P450 from one of the four datasets were annotated as “pseudogenes”. The genome annotation V1 stored in Grape Genome Database hosted at CRIBI ([40]; http://genomes.cribi.unipd.it/DATA/GFF/V1.phase.gff3) and a set of expertized and functional grape cytochromes P450 were used to guide the manual curation.

To validate the gene structure, two transcript datasets were used. First, the *Vitis vinifera* unigene set build #15 from the NCBI database was downloaded (ftp://ftp.ncbi.nih.gov/repository/UniGene/Vitis_vinifera/Vvi.seq.uniq.gz). The 32,193 unigenes were mapped on the PN40024 12X.2 sequence using GMAP version 2013-11-27 [41] using the default parameters except for the format parameter which was set to “gff3_match_cdna”. The second transcript dataset was locally assembled using six RNA-Seq experiments ([42], SRR519450, SRR519456, SRR520380 and SRR520385; [43], all four samples; [44], SRR493740- SRR493746; [45], SRR866544, SRR866570, SRR866571 and SRR866576; [46], SRR522472, SRR522477 and SRR522478; and four RNA-Seq datasets submitted in the frame of this study. The software Tophat2 v2.0.11 [47] was used to map the RNA-Seq reads against the PN40024 12X.2 sequence using the following parameters: -p 5 -N 5—read-edit-dist 5. The software Cufflinks v2.2.1 [48] was used to assemble the transcripts from all the RNA-Seq experiments. First the cufflinks command was used with the -p 5 parameter and then the cuffmerge command with the -p 15 parameter and using the fasta file of the PN40024 12X.2 sequence for the -s parameter. This assembly led to 32,219 transcripts and to a gtf file showing their mapped location in the PN40024 12X.2 sequence. The two transcript datasets were formatted in gff format compatible with the Artemis Browser so that the predicted gene structures of the cytochromes P450 could be compared with the transcripts and edited if needed.

The command maskFastaFromBed v2.19.1 from the bedtools package [49] was used to mask the regions of the PN40024 12X.2 sequence where we annotated cytochrome P450 exons after having reformatted the gff file of the annotations into a bed file. We performed TBLASTN analyses of the four grape cytochrome P450 datasets against the masked PN40024 12X.2 sequence and parsing analyses using the same parameters and cutoffs as previously described. This step allowed identifying the region of the grape genome for which a cytochrome P450 similarity was missed during the manual curation.

To validate the set of complete genes of cytochromes P450 that we annotated, a BLAST against non-redundant sequence database (NR) was performed and only the genes for which the best hit was a cytochrome P450 were kept. For the pseudogenes, a BLASTX was performed against the set of complete P450 genes that we annotated and we kept only the ones that aligned over at least 30% of the query length with the percentage identity of 50%.

The presence of physical clusters of cytochrome P450s in the grape genome was tested based on the following definition of a cluster. Two consecutive P450 annotations are part of a cluster if they are separated by 200kb and 8 non-P450 genes at the most [50,51]. The two annotations also have to be located on the same scaffold, which guaranties a precise estimation of the intergenic distances. A bootstrap test was performed to check whether the cytochromes P450 were more clustered than what is randomly expected. A homemade script was developed with R version 3.0.2 [52]. Ten thousand sampling without replacement of 579 (number of P450 annotations) or 279 features (number of complete P450 genes) were performed on the genome annotation V1 stored in Grape Genome Database hosted at CRIBI counting 29,971 features. The percentage of features organized in clusters was computed using the same protocol as for cytochromes P450. The p-value was calculated by counting each time a percentage equal of greater than the percentage of P450 in clusters divided by 10000 (number of iterations).

Sequence similarity within and between clusters was analyzed by performing a BLASTP search of translated complete P450 genes against themselves. Only the genes that aligned over at least 70% of the query length with the percentage identity of 40% were kept. The Circos software [53] was used to draw the figure. Clusters that contained less than two complete genes were excluded from this analysis (i.e. clusters that contained partial genes, pseudogenes and putative pseudogenes with less than 2 complete genes).

The dotter software version 4.23 [54] was used to draw the sequence similarity graphs of the cluster 190 with its fasta sequence and annotations in a gff format as an input.

### Sequence classification

Cytochrome P450 genes, partial genes and putative pseudogenes were aligned to the P450 sequences from the heterozygous Pinot Noir genome, retrieved from the cytochrome P450 homepage (http://drnelson.uthsc.edu/CytochromeP450.html). In the case of protein sequence identity above 95%, the original name was kept. New sequences were assigned a family based on the best hit among already named grapevine P450s. Twenty-two sequences were given a new CYP name.

### Phylogeny

**Fig 1:**
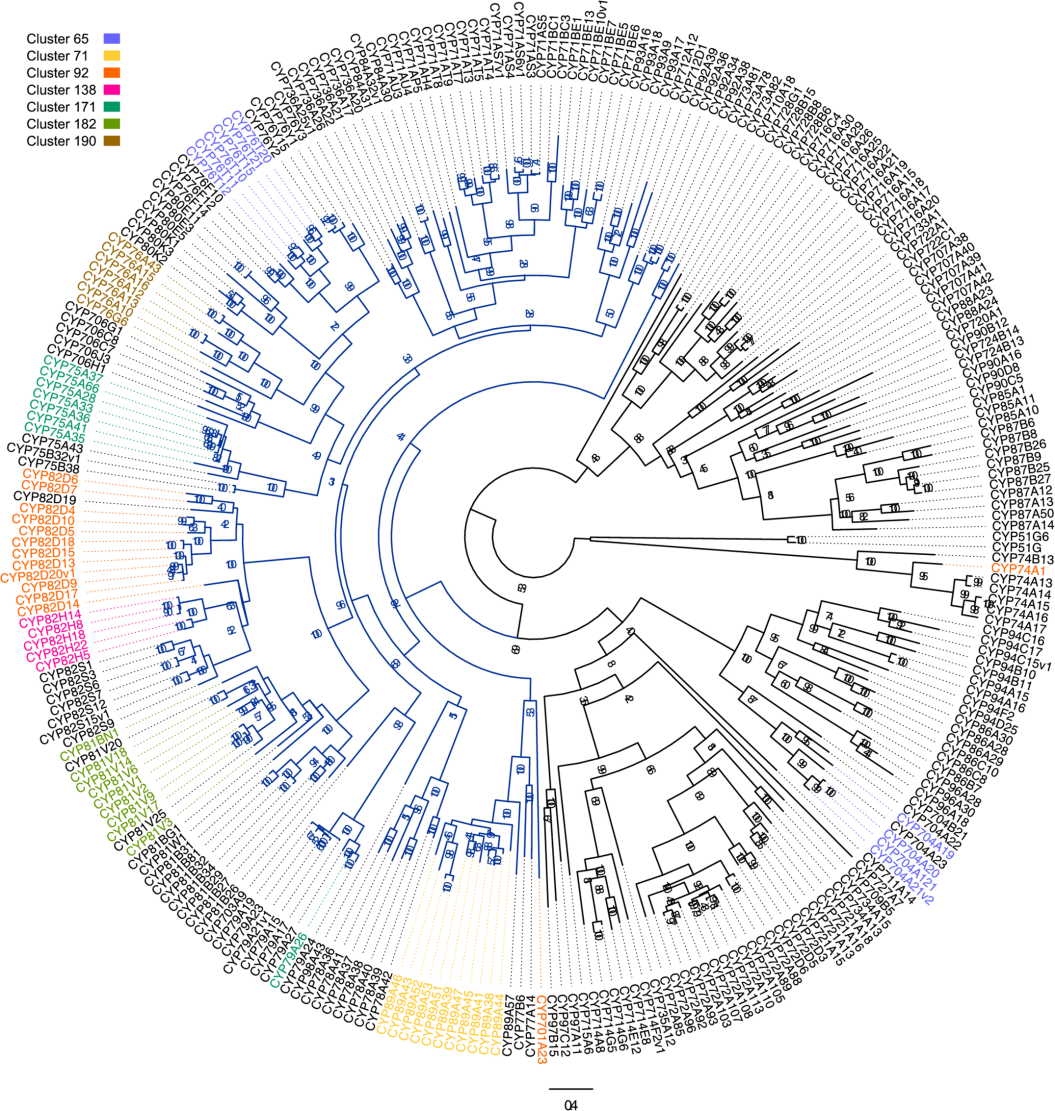
Molecular phylogenetic analysis of grapevine cytochrome P450. The alignment of full-length cytochrome P450 protein sequences was used to generate a maximum likelihood tree. The dark blue clade is the clan 71, which often contains genes involved in specialized metabolism. The highlighted genes belong to the seven largest physical clusters.

Sequences from non-Vitis species were retrieved from the cytochrome P450 homepage (http://drnelson.uthsc.edu/CytochromeP450.html). Pseudogenes and incomplete genes were excluded from the analysis. 279 *Vitis vinifera* CYP (Fig 1) and 191 CYP76, 80 and 706 protein sequences from *Aquilegia caerulea*, *Nelumbo nucifera*, *Mimulus guttatus*, *Solanum lycopersicum*, *Amborella trichopoda*, *Oryza sativa*, *Brachypodium distachyon*, *Arabidopsis thaliana*, *Medicago trunculata*, *Populus trichocarpa* and *Vitis vinifera* (S1 Fig) were aligned with MUSCLE [55] implemented in Seaview [56,57]. Conserved sites were selected in the alignment using Gblocks [58] using the less stringent option parameters. Maximum likelihood phylogenies were obtained from the full-length alignments and from the subset of more conserved sites alignments (all *Vitis* CYP: 166 sites and 11 species CYP alignment: 278 sites) using RAxML (v 8.2.4) [59] via the CIPRES Science Gateway [60] and PhyML (implemented in Seaview v 4.5.4) [61]. Bootstrap values are shown on the nodes of the Vitis all CYP phylogeny. Nodes with bootstrap values below 60 were manually suppressed from the 11 species CYP phylogeny and are shown as trifurcations (unsolved topologies). The trees were visualized and colored using Figtree (http://tree.bio.ed.ac.uk/software/figtree). The species cladogram in (S1 Fig) was inferred from the APGIII system [62].

### Gene expression

We retrieved raw grape RNA-Seq data from NCBI SRA public database (http://www.ncbi.nlm.nih.gov/sra). Fifty-nine sequence files generated in the framework of six different experiments [42,43,45,46,63,64] and four RNA-Seq datasets submitted in the frame of this study were used. The data were formatted in the fastq format using the fastq-dump command from the SRA Toolkit package version 2.3.4 (http://www.ncbi.nlm.nih.gov/books/NBK158900).

Alignments of these reads against the PN40024 12X.2 sequence were then performed using GSNAP version 2013-11-27 [65] with the following parameters: -B 4, -N 1, -n 3,—nofails and the quality protocol according to the experiment. These files were parsed to keep the best, unique and paired (if paired-end reads) alignments using a homemade script.

The number of fragments aligned on each annotation from the genome annotation V1 stored in Grape Genome Database hosted at CRIBI and the cytochromes P450 was counted using the command htseq-count from the HTSeq framework version 0.6.0 [66] with the following parameters: -m intersection-nonempty and -s no. Using a homemade script, FPKMs (Fragments Per Kilo base of exon per Million fragments mapped) were calculated for every annotation.

Using all non-zero FPKM values, the 33th and 66th quantiles were calculated to assign the expression values to one of the four levels of expression chosen: no, low, average and high expression. The experiments were grouped into six categories regarding the conditions under which the samples were obtained. These categories were: leaves, downy mildew (*Plasmopara viticola*) infected leaves, powdery mildew (*Erysiphe necator*) infected leaves, flowers, young berries and ripe berries. An average expression per category was then calculated for each gene and assigned to one of the four levels of expression regarding its value: no expression if the average was zero, low expression between zero and the 33% quantile, average expression between 33% and 66% quantile and high expression for averages higher than the 66% quantile.

The average expression values for each P450 annotation were used to perform a clustering analysis using HCE version 3.5 [67] with a complete linkage method and a Pearson’s correlation as distance measure. The cut-off to define the clusters was set at a Pearson’s correlation coefficient of 0.656. The heatmap was drawn using the package “pheatmap” [68] after row normalization ((FPKM value − row minimum) / row maximum) in R [52].

A RNA-Seq dataset of 48 conditions for berries at four developmental stages (bbch75 = pea size; bbch77 prior to veraison; bbch85 at the end of veraison; bbch89 ripe) for four grapevine varieties (Sangiovese, Barbera, Negro amaro and Refosco) in triplicate, published by Palumbo and coworkers [69] was used to perform an analysis of differentially expressed genes. The reads were aligned using STAR [70] and counted using featureCounts [71] on the grapevine reference genome PN40024 12X.2 and the VCost.v3 annotation [72] supplemented with the cytochrome P450 annotations. The analysis of differentially expressed genes across the four varieties was performed on the whole gene set of the grapevine genome using the script AskoR_DE.R (https://github.com/askomics/askoR) with the parameter cpm > 0.5.

### Accession numbers

RNA-Seq datasets of green and mid-ripening berries from Riesling and Gewurztraminer were submitted at NCBI SRA public database under the BioProject accession number PRJNA378596. PRJNA254035, PRJNA168987, PRJNA244752, PRJNA203687 and PRJNA169607 RNA-Seq BioProjects were retrieved to complete the analysis.

## Results

### Gene annotation, classification and phylogeny

A similarity search of the *V*. *vinifera* PN40024 genome with known P450 sequences revealed 579 putative P450 sequences (S1 File). We manually curated the sequences obtained with a gene prediction algorithm, and validated the annotation with grapevine unigenes and RNAseq reads (see Material and methods). We distributed them into four categories: genes, partial genes, putative pseudogenes and pseudogenes. This led to the identification of 279 full-length genes, which is fewer than the 315 genes reported for the heterozygous Pinot Noir genome on the Cytochrome P450 homepage (http://drnelson.uthsc.edu/CytochromeP450.html), and suggests that some sequences previously annotated as different genes are probably allelic variants. The number of cytochromes P450 in grapevine is comparable to their number in other plants (e.g. 242 in *Arabidopsis thaliana*, 272 in *Solanum lycopersicum* and 309 in *Oryza sativa*). Twenty sequences were annotated as partial genes, lacking a segment of the sequence due to gaps in the genome assembly. Eleven putative pseudogenes only contain one nonsense mutation or frame shift, which could originate from sequencing errors or be genuine but still exist as functional genes in some varieties. Finally, the 269 pseudogenes are fragments, either containing multiple stop codons or frameshift mutations, or sequences not aligning to the whole length of homologous P450 genes.

Grapevine P450s can be assigned to 48 families based on sequence identities. A phylogenetic analysis either of the full-length sequences or of a subset of conserved P450 sites confirmed this classification for most of the families. One exception is CYP90B, which is clustered with CYP720 and CYP724 as previously observed [73]. Other exceptions are the families CYP76 and CYP80, which form a monophyletic group (Fig 1, see Material and methods). We thus investigated the phylogeny of these two families in the broader context of selected angiosperm species (S1 Fig). CYP80 clearly groups with CYP76 sequences, but forms an independent clade between CYP76A/G and the rest of CYP76 sequences (labeled core CYP76). Within the CYP76A/G clade, a eudicot duplication gave rise to the two subfamilies CYP76A and CYP76G. Within the large “core CYP76” clade the uncertain position of both the monocot and *Amborella trichopoda* CYP76s could be due to a problem of long-branch attraction. A specific core eudicot duplication gave rise to CYP76F/B/X on one side and CYP76T/C/E on the other side. These tree topologies were obtained both with the full-length alignment and the partial alignment of conserved sites. Although species-specific “blooms” appeared in the whole CYP76/80 family, they are particularly abundant in the “core CYP76” clade. Different subfamilies expanded in different species.

Comparison of P450 family sizes between species (S2 Fig) allowed us to identify families that expanded in grapevine and might have a role in the production of species-specific specialized metabolites. An expansion of the CYP75 family, involved in anthocyanin biosynthesis, is already well documented [74], whereas the function of CYP82, the largest P450 family in grapevine with 25 members, is currently unknown in this species. Other families that are larger in grapevine than in most other species are: CYP76, CYP79, CYP80, CYP81, CYP87, CYP89 and CYP716.

### Structural organization of the P450s in the PN40024 genome

The 579 cytochrome P450 sequences are distributed on all the 19 chromosomes. Some chromosomes, namely 18, 19 and 6, carry a large number of P450s (77, 57 and 51 sequences, respectively), whereas others, for example chromosome 5, carry very few (8 sequences) (S3 Fig). Twenty-four P450 sequences (7 genes, 6 partial genes, 11 pseudogenes) are located on the “Unknown” chromosome, which is composed of scaffolds that could not be anchored on any of the 19 chromosomes. Since the genome is not completely homozygous (estimated homozygosity is 93% [9]), the “Unknown” chromosome may also contain allelic variants of genes that are placed on the 19 chromosomes.

**Fig 2:**
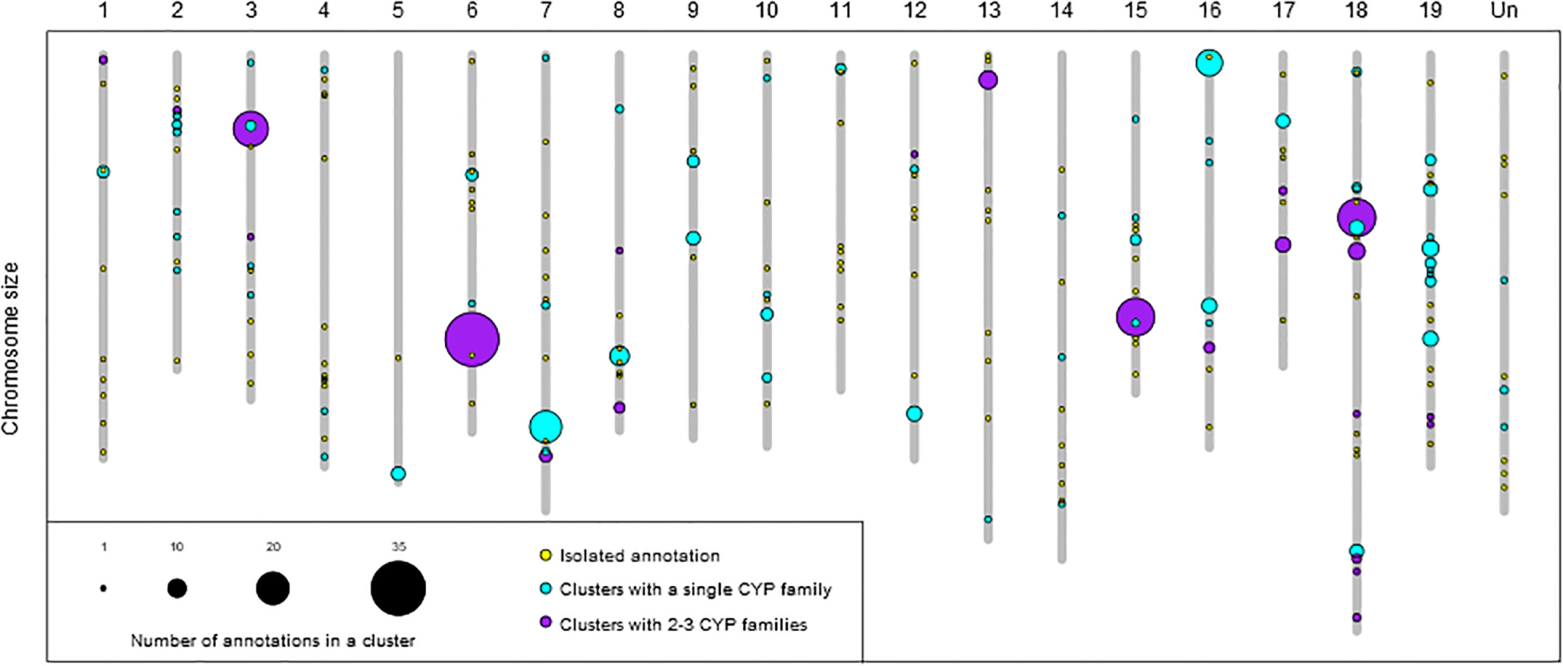
Physical map of cytochrome P450 sequences on the 19 V. vinifera chromosomes. Yellow circles represent isolated annotations, light blue circles represent physical clusters composed of members of only one P450 family and the purple circles represent physical clusters composed of members of 2–3 P450 families. The circle size is proportional to the number of sequences in the cluster. The numbers 1–19 are chromosome numbers and “Un” is “Unknown chromosome” which contains sequences with unknown chromosome location.

We further investigated the distribution of cytochrome P450 sequences in clusters or groups in close physical proximity (separated by less than 200 kb and eight non-P450 genes [50,51]). Our results show that P450 sequences are organized in clusters and not randomly distributed in the genome (bootstrap test, *p*-value < 0.0001). A large majority of cytochrome P450 sequences (452 or 78%) are part of one of the 85 clusters and only 22% (127 P450 sequences) are isolated in the grape genome. The largest number of clusters (40%) are only composed of two P450 sequences, whereas the largest cluster counts thirty-five P450 sequences. On average, there are five P450s per cluster and the median is three P450s per cluster (S4 Fig). The clusters are not enriched neither in complete genes nor pseudogenes, compared to isolated annotations (data not shown). Some chromosomes, such as 16 and 18, are enriched in clustered P450s, whereas others, such as chromosomes 4 and 11, are enriched in isolated P450 (Fig 2 and S3 Fig).

Cytochrome P450 families group genes with higher sequence similarity (≥40% protein sequence identity) and often a similar function. A majority of physical clusters are composed of members of only one P450 family (63 clusters, 74%) and the remaining clusters are composed of up to three P450 families. The four largest clusters are composed of several P450 families, whereas the clusters with single P450 families are smaller (Fig 2 and S4 Fig). Most of the largest P450 families (CYP82, CYP71, CYP81, CYP76, CYP72 and others) are organized in clusters (S1 Table).

**Fig 3:**
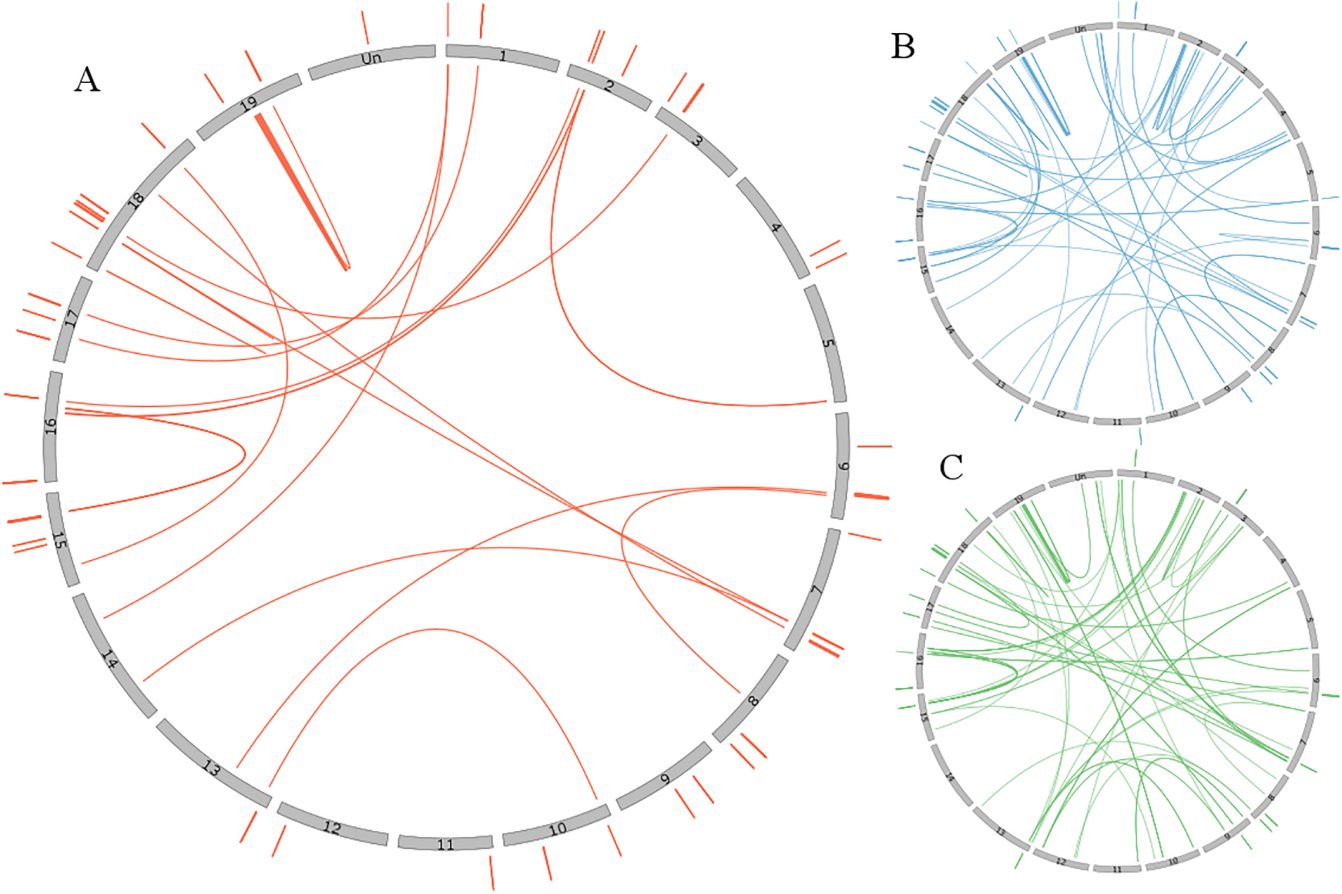
Similarity of the P450 genes between and within clusters. For each circle, the grey bars correspond to the 19 grape chromosomes and the “Unknown chromosome”. The lines connect complete P450 genes according to their similarity. The lines outside the circles show the similarity between genes of the same cluster, whereas the lines in the circle connect similar genes of different clusters. Only P450 genes that form clusters composed of at least two complete genes are illustrated here. The seven largest clusters are labeled with numbers corresponding to Table 1. The lines are connecting the genes corresponding to the best BLAST hit (A), second best hit (B) or third best blast hit (C).

Clustering by P450 family already indicates that more similar P450 sequences cluster in closer physical proximity. But many P450 families are dispersed among several clusters. We thus wished to explore whether the closest paralogs belong to the same or different clusters (Fig 3). The majority of clustered P450 genes (86%) have their closest paralog (the best BLAST hit) in the same cluster. The second and third closest paralogs (second and third best BLAST hit) are in the same cluster for 58% and 49% of the clustered P450 genes. The sequence similarities within the same cluster are thus higher than between clusters.

### Large P450 clusters in *V*. *vinifera* genome formed *via* different mechanisms

To investigate the mechanisms underlying the formation of large groups of physically close cytochrome P450 genes (hereafter called physical clusters), we further analyzed the sequence similarity within clusters, taking into account not only the coding P450 sequences, but also the surrounding non-coding-sequences. This allowed us to infer the mechanism of cluster formation. We focused on the seven largest physical clusters, which comprises between eleven to thirty-five P450 sequences (Table 1). Together, these seven clusters contain 23% of all P450 genes, and a similar fraction of total P450 sequences. Most of the sequences in these clusters are part of “clan 71”, which is a large clade of plant cytochromes P450 often involved in the biosynthesis of species-specific adaptative metabolites (Fig 1).

**Table 1:** Description of the seven largest physical P450 clusters in the *V*. *vinifera* genome.

Label	Chr	Location	Total seq.	Complete genes	Expressed seq.	Co-expression	Organization
65	15	15572751.. 15909327	20 CYP76 4 CYP704	10	20	Flowers and constitutive	Low similarity among members
71	16	401789.. 596606	16 CYP89	11	14	All leaves	Single gene duplications
92	18	9625486.. 9912876	22 CYP82 1 CYP74 1 CYP704	14	16	Powdery mildew infection and ripe berries	Duplicated blocks with co-expression; some single gene duplications
138	3	4387722.. 4512089	22 CYP82	5	16	Young berries	Small duplicated blocks, a few are co-expressed, single gene duplications
171	6	16790972.. 17446396	21 CYP75 14 CYP79	8	28	All leaves and powdery mildew infection	Single gene duplications
182	7	22260680.. 22372250	20 CYP81	9	15	Berries	Small duplicated blocks, a few are co-expressed, single gene duplications
190	8	18038159.. 18121816	11 CYP76	7	9	Flowers	Duplicated blocks with co-expression; some single gene duplication

Label—sequential number of each cluster in the genome; Chr—chromosome number; Location—chromosome coordinates; Total seq.—number of P450 sequences in each cluster, including complete and partial genes, putative pseudogenes and pseudogenes with their family distribution; Complete genes—number of complete P450 genes in the cluster; Expressed sequences—number of expressed P450 sequences in the cluster, co-expression—expression pattern of the cluster; Organization—description of structural organization and mechanism of formation of each cluster.

**Fig 4:**
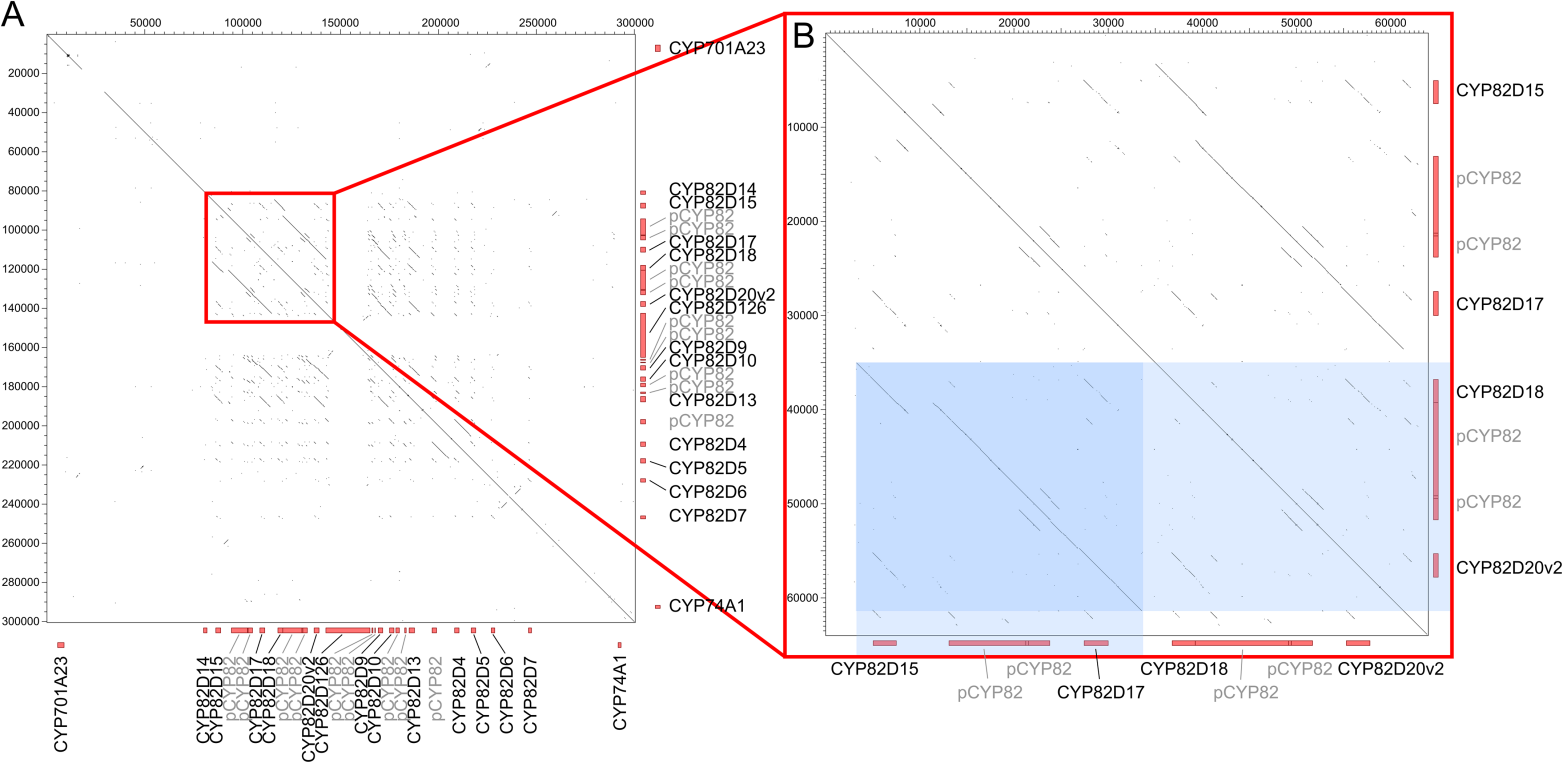
Dot matrix of segmental duplications in the physical cluster 92. Physical cluster 92 is located on chromosome 18 and comprises twenty-two CYP82 sequences, one CYP74 sequence and one CYP704 sequence. The dots and the black lines represent the sequence similarities in cluster **92** compared to itself. The red rectangles on the sides of the graph represent cytochrome P450 sequences. Complete genes are labeled with their name and pseudogenes are labeled with “p” and the P450 family. A) The similarities for the whole cluster **92**. B) A zoom of the red squared region, which contains two 30kb blocks with very high similarity. Analysis of gene expression showed that CYP82D15 and CYP82D18 are co-expressed (expression cluster **A**, expression in ripe berries) as well as CYP82D17 and CYP82D20v2 (expression cluster **C**, expression in downy mildew infected leaves). The pseudogenes of the enlarged segment are not expressed.

Analysis of similarity blocks within these clusters showed they differ remarkably in their structures (S5 Fig). One of the largest physical clusters, cluster 65, is characterized by low similarities, both among the P450 sequences and surrounding non-coding regions. The similarity blocks of two other large physical clusters, 71 and 171, are restricted to P450 sequences and do not extend to the intergenic regions. Single gene duplications were thus probably the main mechanism of formation of these two clusters. The similarity blocks of physical clusters 138 and 182 extend to the non-coding regions around the cytochromes P450 annotations. This suggests the duplication events leading to formation of these clusters happened relatively recently. High similarity between the non-coding regions, which include the promoter regions, should result in similar expression profiles. Cluster 138 has the highest fraction (73%) of pseudogenes of all the seven large clusters. In physical clusters 92 and 190, the similarity blocks extend over even longer regions that include three to four cytochrome P450 sequences and their intergenic regions (Fig 4). In addition, the type of annotation (gene or pseudogene) was also maintained in the same order between duplicated blocks. This suggests these two clusters formed through very recent proximal segmental duplications.

### Expression profiles of grapevine P450s

To identify P450 genes with potential roles in pathogen resistance or biosynthesis of berry metabolites we analyzed the expression of the 579 P450 sequences. Pseudogenes were included in the analysis of expression to account for recently pseudogenized sequences that may still be expressed to some extent. We used 59 RNA-Seq datasets (S2 Table), which describe gene expression in different tissues (flowers, berries, leaves), different stages of berry development, and pathogen infection (downy and powdery mildews). To enable a meaningful comparison of gene expression between different experiments we calculated fragment number per kilobase of transcript per million mapped reads (FPKM) for each P450 sequence (S1 File). The majority of P450 sequences (457 or 79%) were expressed in at least two experiments. Of the remaining 122 non-expressed P450 sequences, only seven were complete genes. Out of the expressed P450 sequences, complete genes showed higher expression (mean FPKM = 10.6, median FPKM = 0.4) compared to pseudogenes and putative pseudogenes (mean FPKM = 2.4, median FPKM = 0) or partial genes (mean FPKM = 1.7, median = 0.1).

To simplify the dataset, we grouped the 59 experiments in six categories: leaves, downy mildew (*Plasmopara viticola*) infected leaves, powdery mildew (*Erysiphe necator*) infected leaves, flowers, young berries and ripe berries. “Flowers”, “young” and “ripe berries” categories were significantly enriched in expressed P450 sequences whereas “leaves” and “downy mildew” categories were depleted (chi^2^ test, p-value = 1E-15). In addition, we grouped the expression levels into four classes (no, low, medium or high expression). The “powdery mildew” category was found to be significantly enriched for highly expressed P450 sequences whereas the “leaves” category was depleted (chi^2^ test, p-value = 9E-16). Altogether, these results indicate a significant induction of P450 expression caused by biotic stress, especially powdery mildew infection, and in grapevine organs synthesizing aromas and volatile compounds.

**Fig 5:**
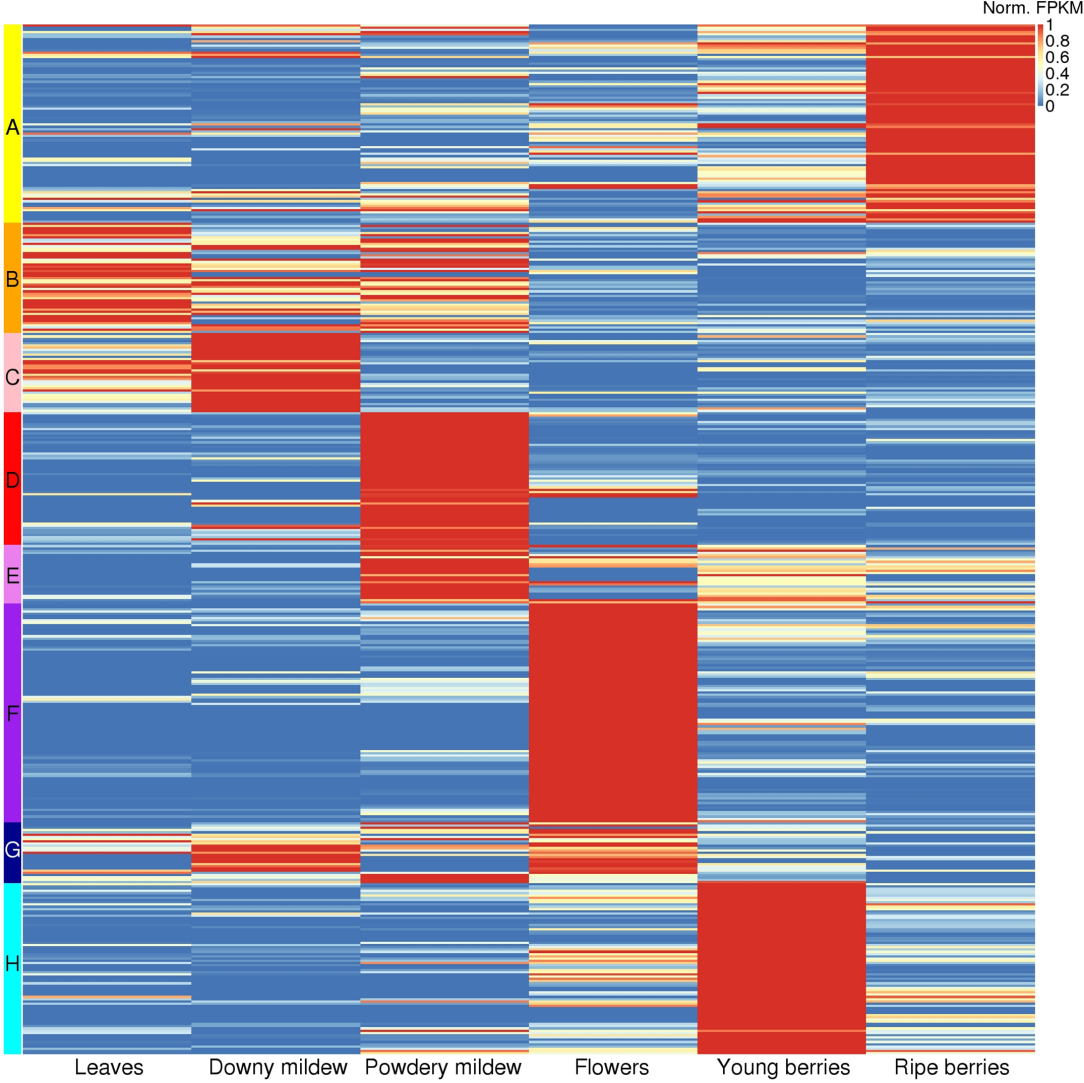
Heatmap of the P450 sequences, clustered according to their expression profile. The expression levels were averaged over the experiments classified in one of the six experimental categories: leaves, downy mildew (*Plasmopara viticola*) infected leaves, powdery mildew (*Erysiphe necator*) infected leaves, flowers, young berries and ripe berries. This heatmap includes the 457 expressed cytochrome P450 sequences. The color scale for the expression level represents FPKM values normalized by row ((FPKM value − row minimum) / row maximum). The color bars on the left are showing the eight expression clusters, which are designated by the letters on the side.

We analyzed the expression patterns by clustering the expression profiles of the 457 expressed cytochrome P450 sequences. A Pearsons’ correlation coefficient cut-off of 0.656 resulted in eight expression clusters, shown in Fig 5. Only twenty-seven P450 sequences (6%, grouped in expression cluster G) are expressed constitutively, that means expressed at similar levels over the six categories, but the vast majority of the P450 genes are expressed in specific organs or under particular conditions. Among constitutively expressed genes, the CYP76 family was significantly enriched (chi^2^ test, p-value = 3E-7).

A major shift in P450 expression pattern occurs during berry development. Indeed, the three largest expression clusters F (97 sequences), A (88 sequences) and H (76 sequences) grouped P450 sequences preferentially expressed in flowers, ripe berries and young berries, respectively. Among the large CYP families, CYP76, CYP716 and CYP714 were significantly enriched in cluster F whereas CYP72 was significantly depleted. CYP716 was also enriched in cluster H. CYP78 and CYP80 were significantly enriched in cluster A. In addition, this cluster featured the P450 gene with the highest expression in all experiments: CYP78A41 with an average FPKM value in ripe berries of 1292. Beside this expression analysis on multiple organs of the plant, an analysis of differentially expressed genes during berries development (four stages) for four grapevine varieties was performed using previously published data [70]. Two hundred and forty five P450 genes were significantly differentially expressed for at least one variety (S6 Fig). Notably, CYP76, CYP82, CYP71, CYP72, CYP75, CYP81 and CYP89 families that are enlarged in the grapevine genome and/or that are involved in secondary metabolism showed the greatest number of differentially expressed genes (20, 19, 19, 18, 18, 14 and 13, respectively). This result strengthens the hypothesis that these cytochrome P450 gene families play a major role in the biosynthesis of aroma compounds and especially the varietal-specific aromas.

The analysis of gene expression on multiple organs of grapevine also highlighted some P450 sequences with potential role in biotic stress response. Indeed, cluster D (59 sequences) and cluster C (35 sequences) grouped P450 sequences preferentially expressed during powdery and downy mildew infections, respectively. Among the large CYP families, CYP82, CYP87 and CYP736 were significantly enriched in cluster D whereas CYP72 was significantly enriched in cluster C.

We more thoroughly investigated co-expression of cytochrome P450 sequences within the physical clusters. We found that 245 out of the 343 expressed P450 sequences that are part of a physical cluster (71%) show an expression profile similar to at least one member of the same physical cluster. This level of co-expression is highly significant (bootstrap test, p-value<0.0001), which means that P450 physical clusters are highly enriched in co-expressed sequences. The co-expression was analyzed further in the seven largest physical clusters to check whether tandem duplicated genes observed in the previous section maintained similar expression profiles (Table 1). Especially, we identified four large clusters with high similarity, not only among the coding, but also the non-coding regions. These non-coding regions presumably include promoter sequences, so the genes in these clusters are expected to have the same expression pattern. Indeed, the 30 kb duplicated block within cluster 92 (Fig 4) retained the same expression profile after the segmental duplication. The duplicated segment consists of four P450 sequences: two genes and two pseudogenes. The two pseudogenes in both blocks were not expressed, whereas the two complete genes in both blocks (CYP82D15 and CYP82D18; CYP82D17 and CYP82D20v2) were co-expressed in ripe berries (cluster A) and downy mildew infected leaves (cluster C), respectively. Six out of sixteen expressed sequences in this cluster show induction in powdery mildew infected leaves (cluster D), whereas five other P450 sequences in the same cluster were up-regulated in ripe berries (cluster A). Interestingly, cluster 138 is also composed of CYP82 sequences, but these sequences were preferentially expressed in young berries (cluster H). Eight out of nine expressed CYP76 sequences in the physical cluster 190, on the other hand, were co-expressed in flowers (cluster F). In cluster 71, five out of fourteen expressed CYP89 genes were specifically expressed in leaves (cluster B). Cluster 182 grouped ten out of fifteen expressed CYP81 sequences specifically expressed in berries (5 sequences in cluster A and 5 sequences in cluster H).

## Discussion

We produced an exhaustive, reliable and validated manual annotation of cytochromes P450 in the genome of the nearly homozygous grapevine (*V*. *vinifera)* accession PN40024 [9]. Cytochrome P450 superfamily in *Vitis vinifera* contains both very similar and very divergent genes (sequence identity ranges from 10% to almost 100%), and often form clusters in very close physical proximity, which makes it challenging for automated annotation algorithms. Manual curation is therefore necessary to produce a reliable annotation, suitable for demanding downstream applications such as phylogenetic or gene expression analysis. Grapevine P450s have been previously manually annotated (Cytochrome P450 homepage, http://drnelson.uthsc.edu/vitis.htm) in the highly heterozygous genome of Pinot noir cultivar [33]. Our annotation represents an improvement over the existing dataset for several reasons. The assembly of PN40024 genome is of better quality compared to the Pinot noir genome: it contains fewer gaps and a higher fraction of anchored contigs. The homozygosity of the genome not only enabled a better quality of the assembly, but also assured that most of the annotated sequences are individual loci and not allelic variants. This can partially explain a lower number of cytochrome P450 genes in our annotation—279—compared to the 315 genes reported on the Cytochrome P450 homepage. Additionally, the annotation on the Cytochrome P450 homepage classifies the sequences in only two categories, genes and pseudogenes, whereas we employed a more stringent classification into genes, partial genes, putative pseudogenes and pseudogenes. Lastly, we report the exact genomic coordinates of the P450 sequences, which facilitate comparison to annotations of other genes, and provide insights into structural organization of the grapevine CYPome.

Several gene families involved in the biosynthesis of specialized metabolites, such as terpene synthase and stilbene synthase genes, have expanded in grapevine genome compared to other species [9,75,76]. Although the total number of cytochrome P450 genes in grapevine is comparable to other species, individual P450 families experienced similar expansions. These expanded P450 families, similarly to terpene and stilbene synthases, form large physical clusters of more than ten homologous sequences. One of such families is CYP75, which together with CYP79 family members forms the largest physical cluster of thirty-five P450 sequences on chromosome 6. Expansion of CYP75 genes in grapevine was previously documented, but the presence of another P450 family, CYP79, in the same cluster was not reported [74]. Clustered genes with low or no homology sometimes participate in the same biosynthetic pathway [77,78], but this is unlikely in the case of CYP75 and CYP79, since both families have well established roles in different biosynthetic pathways: CYP79 genes code for amino acid *N*-hydroxylases [27] for the synthesis of oxime derivatives precursors of cyanogenic glucosides, whereas CYP75A genes code for flavonoid 3’,5’-hydroxylases [79], crucial enzymes in the biosynthesis of blue anthocyanins in the grape skin [74,80]. However, we cannot exclude the recruitment of some of these genes in other pathways. Interestingly, the sequencing of the genome of the grapevine cultivar Tannat, characterized by its very deep color, revealed an even higher number of CYP75 genes compared to the PN40024 accession [45]. Copy number of genes in a cluster can therefore vary between cultivars and could influence varietal characteristics. Other expanded P450 families in the grapevine genome are CYP82, CYP76, CYP81 and CYP89. They are forming large clusters resulting from gene duplication, a mechanism proven to favor emergence of new metabolic functions, especially for P450 genes [81].

Large-scale analysis of gene expression across several tissues and conditions provides a first hint to the putative P450 functions in grapevine. Pathogen infection causes a major shift in the P450 expression, inducing members from families CYP736, CYP81, CYP82 and CYP87. Their homologs in other species have been shown to participate in biosynthesis of highly specialized defense compounds (S1 Table). Interestingly, CYP736A25v1, which was shown to be upregulated upon infection with the Pierce disease pathogen *Xylella fastidiosa* [82], is also upregulated upon infection with powdery mildew and downy mildew pathogens. Two other sequences from the CYP736 family are also induced by biotic stress but their expression level is lower. Another large shift in expression occurs in developing grape berries. The most upregulated P450 families in the ripe berries expression cluster are CYP81, CYP82 and CYP78. Along with CYP76, CYP71, CYP72, CYP75 and CYP89, they are also differentially expressed across grapevine varieties during berry development. Therefore, these P450s are likely to participate in the biosynthesis of defense compounds or compounds important for the organoleptic properties of wine (aroma, colour, taste, mouthfeel).

The P450 gene with the overall highest expression level and the most up-regulated P450 gene in ripe berries is CYP78A41. A member of the same P450 family in tomato (*S*. *lycopersicum*) was selected during domestication to increase fruit size [24]. High expression of CYP78A41 in grape berries points to a similar event in grapevine domestication.

The most striking result in our analysis probably concerns the CYP76 family. From a structural point of view, we confirmed that CYP76 family is expanded in grapevine reference genome compared to most of the other plant genomes [35]. Furthermore, the CYP76 family was found to be highly clustered and part of the largest cluster, cluster190, in which a recent tandem duplication involving three consecutive genes was identified. This suggests an active evolution dynamics for this particular CYP family which could favor emergence of new metabolic functions [81] in particular grapevine varieties. We also found that the expression of the CYP76 family was mostly upregulated in grapevine flowers, similarly to what Boachon and coworkers [21] found in *Arabidopsis*. Interestingly, grapevine flowers, just like berries, are very rich in terpenes, some of the most important volatile compounds contributing to the floral bouquet of wine [75,83,84] and CYP76s were already found to be involved in terpene biosynthesis in *Arabidopsis* and grapevine [21,31,85,86]. More, CYP76 family showed the greatest number of genes with varietal-specific expression patterns during berry development. Altogether, its evolutionary dynamics along with its role in terpene pathway make the CYP76 genes major candidates to understand the diversity of grapevine and wine aromas, as recently shown for CYP76F14, a member of CYP76 family, which was identified as a key player in the production of wine lactone, a typical aroma of aged wines [25].

## Conclusions

The phylogenetic and structural data suggest that some P450 families underwent multiple tandem or segmental duplications, which resulted in large physical clusters of homologous sequences that are often co-expressed. Most of these P450 families are involved in biosynthesis of highly specialized metabolites in other plant species. These genes are often expressed in specific conditions and tissues, such as leaves upon pathogen infection or during berry development. Finally, our work provides an exhaustive and robust annotation including clear identification, structural organization, evolutionary dynamics and expression patterns for the grapevine cytochrome P450 families, paving the way to efficient functional characterization of genes involved in grapevine defense pathways and aroma biosynthesis. Especially, our study points out towards the CYP76 family as the key candidate for further understanding the extraordinary diversity of grape and wine aromas.

## Supporting information

S1 Fig Phylogeny of CYP80 and CYP76 in angiosperms. Maximum likelihood tree of full length CYP76 and CYP80 protein sequences from a selection of angiosperms, rooted with CYP706 from all the included species. Nodes with bootstrap values below 60 are collapsed to trifurcations. Species specific clades with more than two members (except *V*. *vinifera*) are collapsed to triangles. The label of the triangle gives the subfamily and the number of members contained in the clade.
(PDF)

S2 Fig Comparison of the number of P450 genes per family between species. Dot size is proportional to the relative family size (number of genes per family) in a given species compared to *Vitis vinifera* (Vv = *Vitis vinifera*, Nn = *Nelumbo nucifera*, Os = Oryza sativa, Bd = *Brachypodium distachyon*, Sl = *Solanum lycopersicum*, At = *Arabidopsis thaliana*, Pt = *Populus trichocarpa*, Gm = *Glycine max*, Mt = *Medicago truncatula*). The numbers in the first column are the absolute family sizes (numbers of genes per family) in *Vitis vinifera*. The number of genes per family was retrieved from the cytochrome P450 homepage. Pseudogenes and families not present in *V*. *vinifera* (CYP83, CYP99, CYP702, CYP705, CYP708, CYP718 and CYP729) were excluded from the count.
(PDF)

S3 Fig Distribution of the *V*. *vinifera* P450s per chromosome. The blue bar corresponds to clustered annotations and the yellow bar to the isolated annotations. The “Unknown chromosome” is labeled as “Un”.
(PDF)

S4 Fig Distribution of the P450 sequences per physical cluster. Median and average values are labeled with arrows. The clusters composed of a single P450 family are represented in blue and those composed of 2 or 3 P450 families in orange.
(PDF)

S5 Fig Dot matrix plots of the largest physical clusters. The dots and the black lines represent the sequence similarities in cluster 92 compared to itself. The red rectangles on the sides of the graph represent cytochrome P450 sequences. Complete genes are labeled with their name and pseudogenes are labeled with “p” and the P450 family.
(PDF)

S6 Fig Heatmap of the differentially expressed cytochromes P450 between berries of four grapevine varieties. Expression in berries at four developmental stages (75 = pea size; 77 = prior to veraison; 85 = at the end of veraison; 89 = ripe) for four grapevine varieties (Sangiovese, Barbera, Negro amaro and Refosco) was studied. This heatmap shows the expression profiles of the 245 differentially expressed cytochromes P450 for at least one variety. The expression levels were averaged over the three replicates for each condition. The color scale for the expression level represents RPKM values normalized by row ((RPKM value − row minimum) / row maximum). The dendrogram on the left shows the clustering by gene. The raw data were obtained from [69].
(PDF)

S1 Table List of P450 families with majority of its members grouped in physical clusters. (PDF)

S2 Table Description of RNA-Seq experiments used for analysis of gene expression. (PDF)

S1 File Fasta file of P450 cDNA, fasta file of P450 proteins, annotation file of P450s in gff3 format, FPKM of P450s in 59 experiments, RPKM for the analysis of differentially expressed P450 in the berries of four varieties, contrasts for the analysis of differentially expressed genes. (XLSX)
