# Supplementary material for: Annotation, classification, genomic organization and expression of the Vitis vinifera CYPome
Source: PLoS One. 2018 Jun 28;13(6):e0199902. doi: 10.1371/journal.pone.0199902 (PMC6023221; doi:10.1371/journal.pone.0199902)
Supplement: S1 Fig — Maximum likelihood tree of full length CYP76 and CYP80 protein sequences from a selection of angiosperms, rooted with CYP706 from all the included species. Nodes with bootstrap values below 60 are collapsed to trifurcations. Species specific clades with more than two members (except V. vinifera) are collapsed to triangles. The label of the triangle gives the subfamily and the number of members contained in the clade. (PDF) [file pone.0199902.s001.pdf]

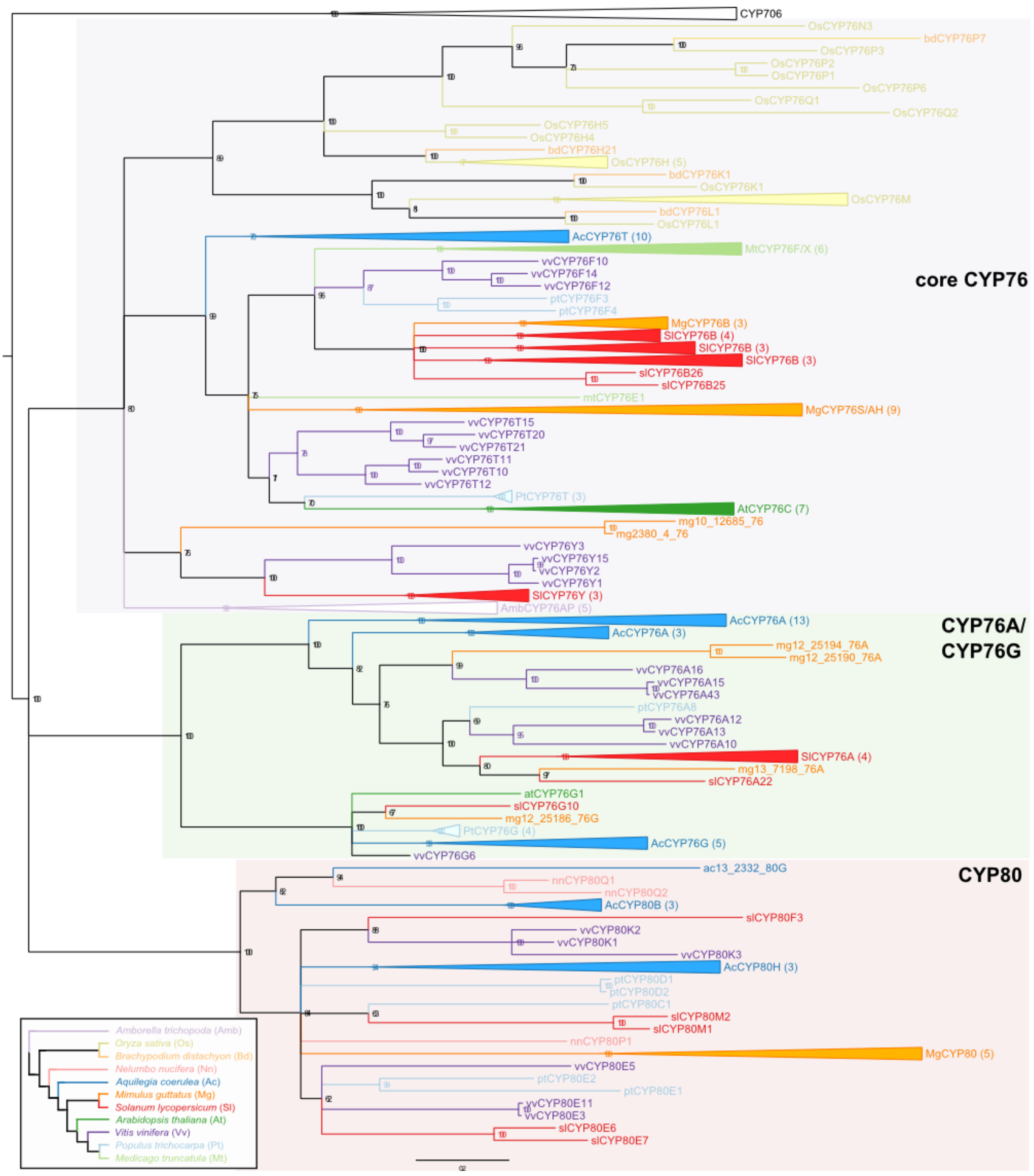

S1 Fig. Phylogeny of CYP80 and CYP76 in angiosperms. Maximum likelihood tree of full length CYP76 and CYP80 protein sequences from a selection of angiosperms, rooted with CYP706 from all the included species. Nodes with bootstrap values below 60 are collapsed to trifurcations. Species specific clades with more than two members (except *V. vinifera*) are collapsed to triangles. The label of the triangle gives the subfamily and the number of members contained in the clade.
