# Supplementary material for: Annotation, classification, genomic organization and expression of the Vitis vinifera CYPome
Source: PLoS One. 2018 Jun 28;13(6):e0199902. doi: 10.1371/journal.pone.0199902 (PMC6023221; doi:10.1371/journal.pone.0199902)
Supplement: S2 Fig — Dot size is proportional to the relative family size (number of genes per family) in a given species compared to Vitis vinifera (Vv = Vitis vinifera, Nn = Nelumbo nucifera, Os = Oryza sativa, Bd = Brachypodium distachyon, Sl = Solanum lycopersicum, At = Arabidopsis thaliana, Pt = Populus trichocarpa, Gm = Glycine max, Mt = Medicago truncatula). The numbers in the first column are the absolute family sizes (numbers of genes per family) in Vitis vinifera. The number of genes per family was retrieved from the cytochrome P450 homepage. Pseudogenes and families not present in V. vinifera (CYP83, CYP99, CYP702, CYP705, CYP708, CYP718 and CYP729) were excluded from the count. (PDF) [file pone.0199902.s002.pdf]

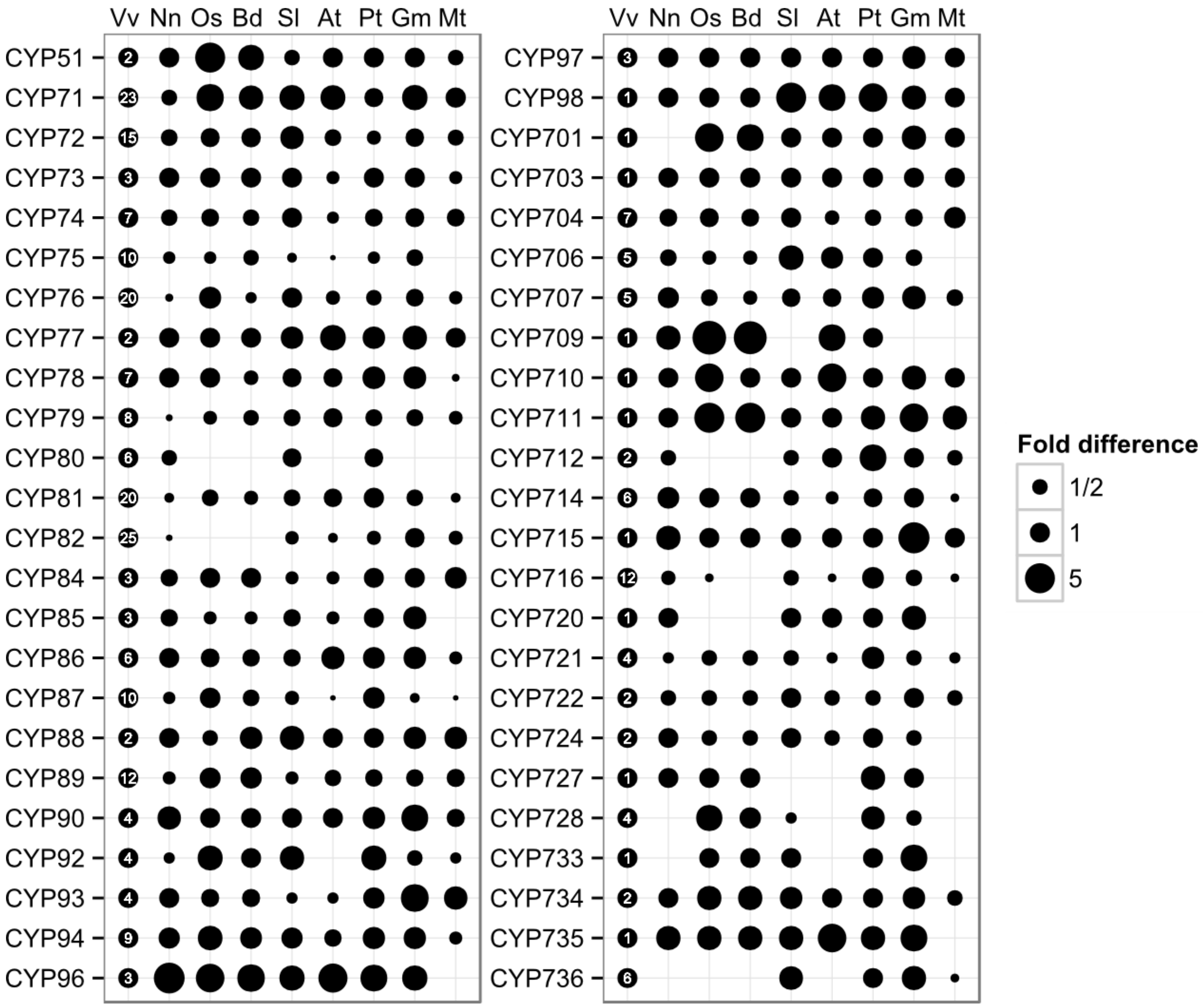

S2 Fig. Comparison of the number of P450 genes per family between species. Dot size is proportional to the relative family size (number of genes per family) in a given species compared to *Vitis vinifera* (Vv = *Vitis vinifera*, Nn = *Nelumbo nucifera*, Os = Oryza sativa, Bd = *Brachypodium distachyon*, Sl = *Solanum lycopersicum*, At = *Arabidopsis thaliana*, Pt = *Populus trichocarpa*, Gm = *Glycine max*, Mt = *Medicago truncatula*). The numbers in the first column are the absolute family sizes (numbers of genes per family) in *Vitis vinifera*. The number of genes per family was retrieved from the cytochrome P450 homepage. Pseudogenes and families not present in *V. vinifera* (CYP83, CYP99, CYP702, CYP705, CYP708, CYP718 and CYP729) were excluded from the count.
