# Supplementary material for: Annotation, classification, genomic organization and expression of the Vitis vinifera CYPome
Source: PLoS One. 2018 Jun 28;13(6):e0199902. doi: 10.1371/journal.pone.0199902 (PMC6023221; doi:10.1371/journal.pone.0199902)
Supplement: S3 Fig — The blue bar corresponds to clustered annotations and the yellow bar to the isolated annotations. The “Unknown chromosome” is labeled as “Un”. (PDF) [file pone.0199902.s003.pdf]

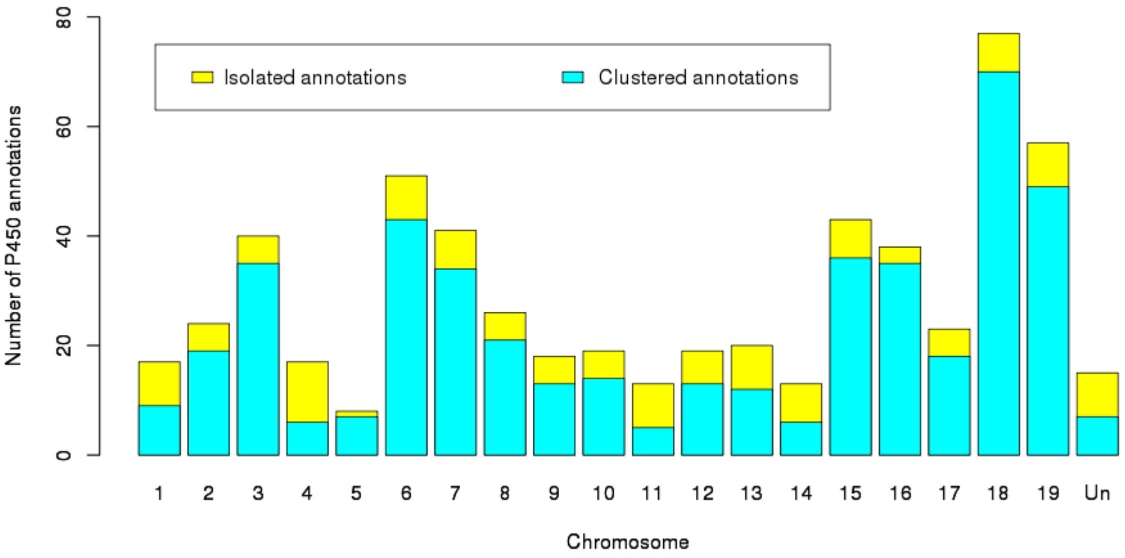

S3 Fig. Distribution of the *V. vinifera* P450s per chromosome. The blue bar corresponds to clustered annotations and the yellow bar to the isolated annotations. The “Unknown chromosome” is labeled as “Un”.
