# Supplementary material for: Annotation, classification, genomic organization and expression of the Vitis vinifera CYPome
Source: PLoS One. 2018 Jun 28;13(6):e0199902. doi: 10.1371/journal.pone.0199902 (PMC6023221; doi:10.1371/journal.pone.0199902)
Supplement: S4 Fig — Median and average values are labeled with arrows. The clusters composed of a single P450 family are represented in blue and those composed of 2 or 3 P450 families in orange. (PDF) [file pone.0199902.s004.pdf]

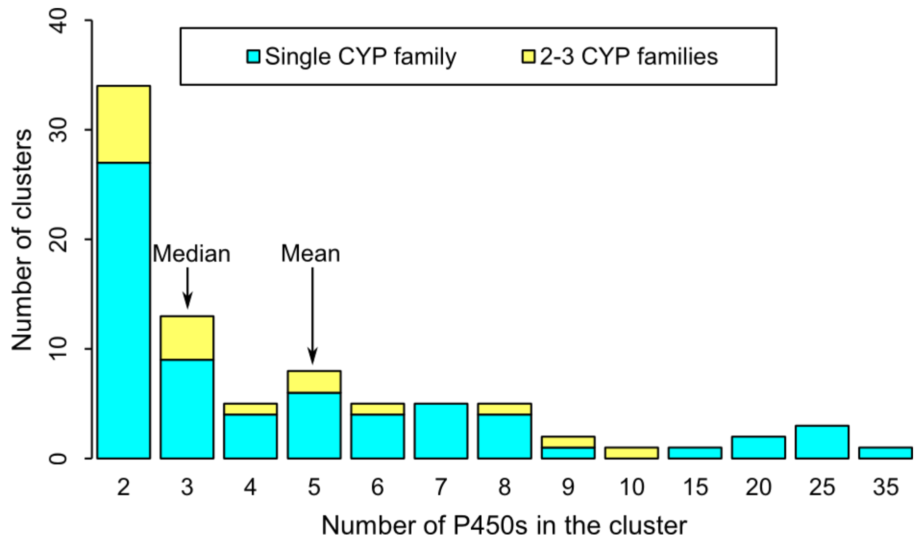

S4 Fig. Distribution of the P450 sequences per physical cluster. Median and average values are labeled with arrows. The clusters composed of a single P450 family are represented in blue and those composed of 2 or 3 P450 families in orange.
