# Supplementary material for: Annotation, classification, genomic organization and expression of the Vitis vinifera CYPome
Source: PLoS One. 2018 Jun 28;13(6):e0199902. doi: 10.1371/journal.pone.0199902 (PMC6023221; doi:10.1371/journal.pone.0199902)
Supplement: S5 Fig — The dots and the black lines represent the sequence similarities in cluster 92 compared to itself. The red rectangles on the sides of the graph represent cytochrome P450 sequences. Complete genes are labeled with their name and pseudogenes are labeled with “p” and the P450 family. (PDF) [file pone.0199902.s005.pdf]

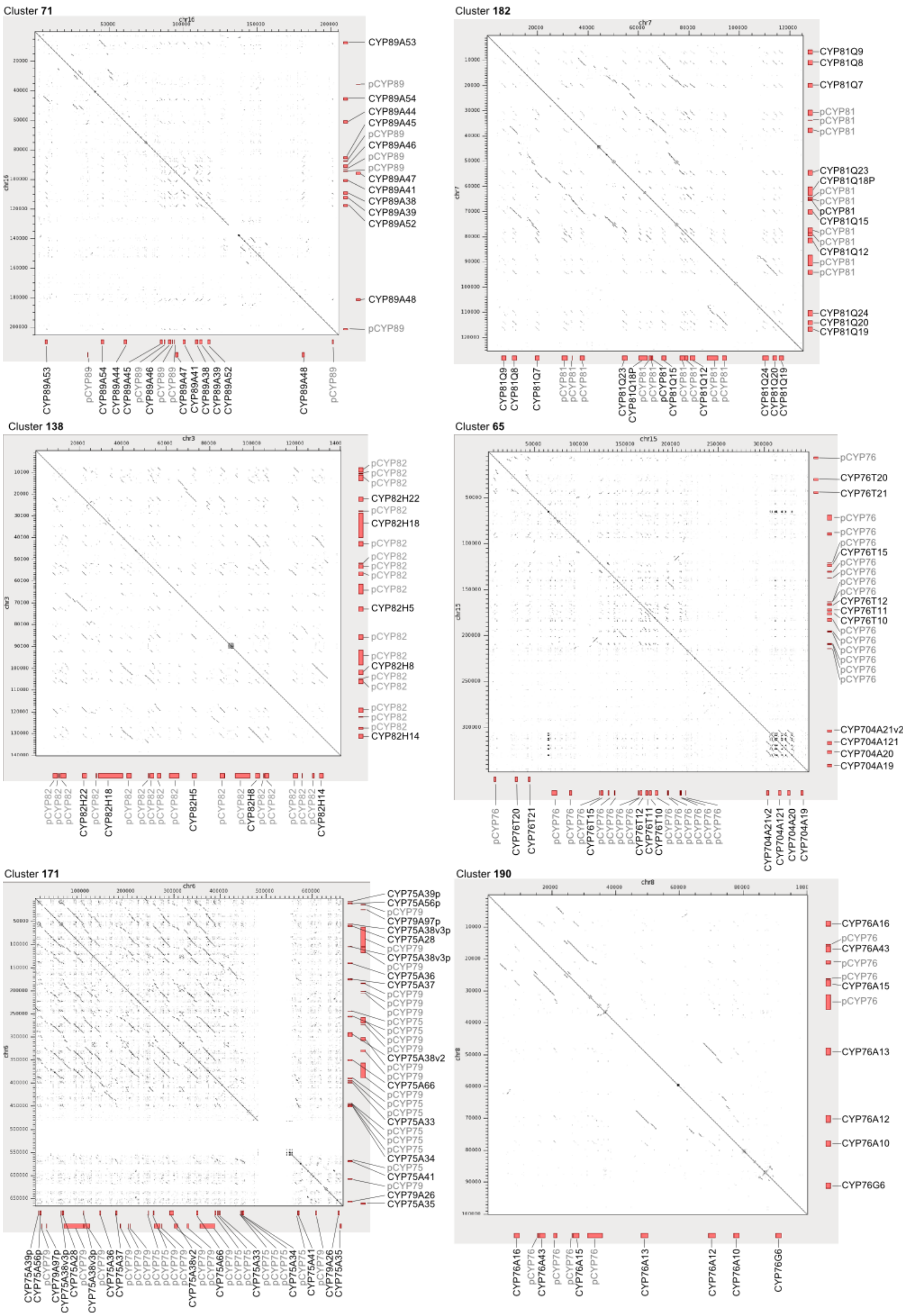

S5 Fig. Dot matrix plots of the largest physical clusters. The dots and the black lines represent the sequence similarities in cluster 92 compared to itself. The red rectangles on the sides of the graph represent cytochrome P450 sequences. Complete genes are labeled with their name and pseudogenes are labeled with "p" and the P450 family.
