# Supplementary material for: Annotation, classification, genomic organization and expression of the Vitis vinifera CYPome
Source: PLoS One. 2018 Jun 28;13(6):e0199902. doi: 10.1371/journal.pone.0199902 (PMC6023221; doi:10.1371/journal.pone.0199902)
Supplement: S6 Fig — Expression in berries at four developmental stages (75 = pea size; 77 = prior to veraison; 85 = at the end of veraison; 89 = ripe) for four grapevine varieties (Sangiovese, Barbera, Negro amaro and Refosco) was studied. This heatmap shows the expression profiles of the 245 differentially expressed cytochromes P450 for at least one variety. The expression levels were averaged over the three replicates for each condition. The color scale for the expression level represents RPKM values normalized by row ((RPKM value − row minimum) / row maximum). The dendrogram on the left shows the clustering by gene. The raw data were obtained from [69]. (PDF) [file pone.0199902.s006.pdf]

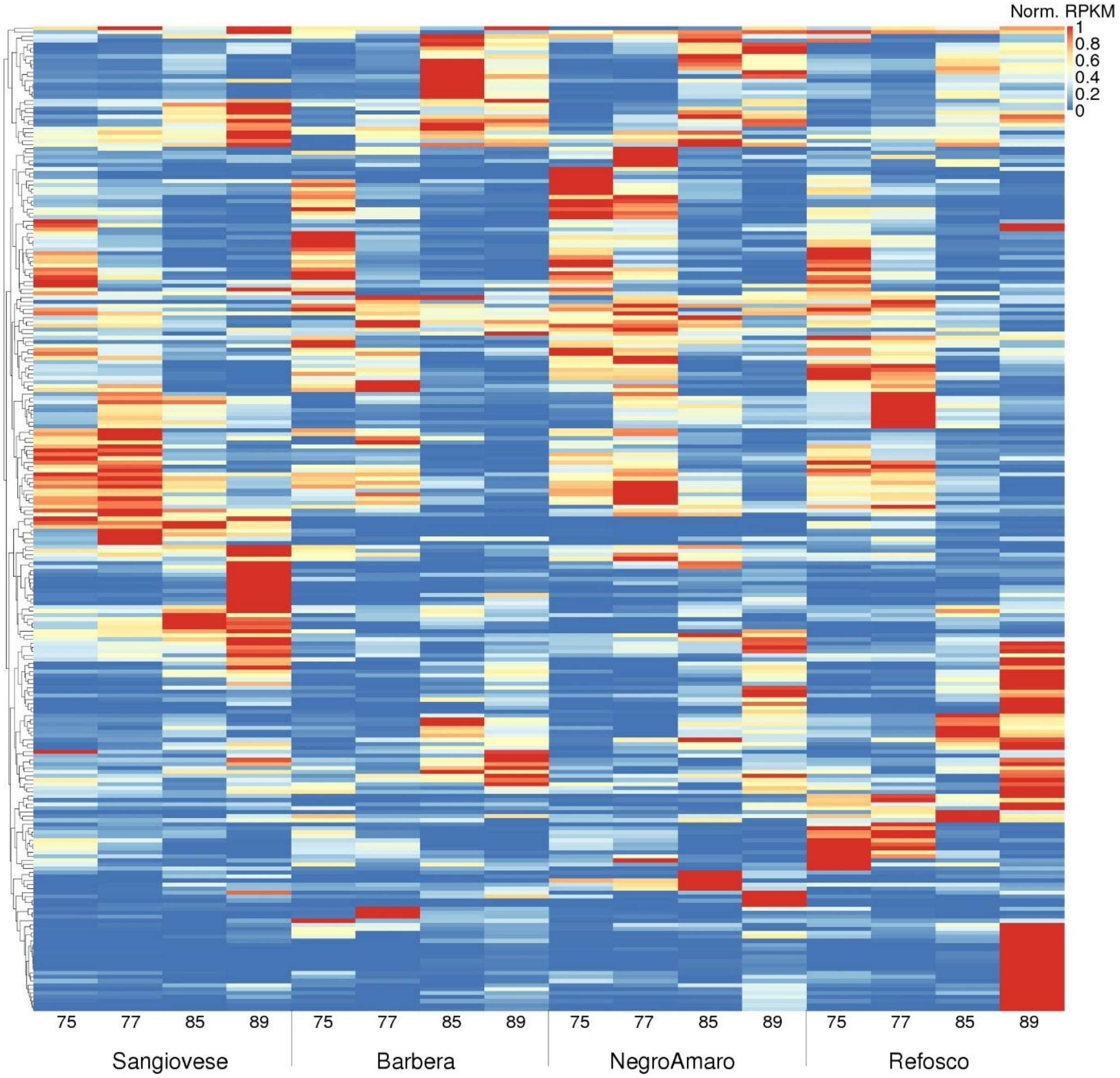

S6 Fig. Heatmap of the differentially expressed cytochromes P450 between berries of four grapevine varieties. Expression in berries at four developmental stages (75 = pea size; 77 = prior to veraison; 85 = at the end of veraison; 89 = ripe) for four grapevine varieties (Sangiovese, Barbera, Negro amaro and Refosco) was studied. This heatmap shows the expression profiles of the 245 differentially expressed cytochromes P450 for at least one variety. The expression levels were averaged over the three replicates for each condition. The color scale for the expression level represents RPKM values normalized by row ((RPKM value – row minimum) / row maximum). The dendrogram on the left shows the clustering by gene. The raw data were obtained from [69].
