# Supplementary material for: Annotation, classification, genomic organization and expression of the Vitis vinifera CYPome
Source: PLoS One. 2018 Jun 28;13(6):e0199902. doi: 10.1371/journal.pone.0199902 (PMC6023221; doi:10.1371/journal.pone.0199902)
Supplement: S1 Table — (PDF) [file pone.0199902.s007.pdf]

S1 Table. List of P450 families with majority of its members grouped in physical clusters.

Family	Family size (total sequences)	Number of clustered members	Examples of functions in other organisms
CYP82	69	66	Biosynthesis of homoterpenes in *A. thaliana* [1], opioids in *Papaver somniferum* [2–4].
CYP71	51	45	Biosynthesis of monoterpenoids in mint species *Mentha x piperita* and *Mentha x spicata* [5], cyanogenic glucosides in cassava (*Manihot esculenta*) [6], furanocoumarins in several species [7], artemisinin in *Artemisia annua* [8], flavonoids in soybean (*Glycine max*) [9].
CYP81	50	42	Biosynthesis of indole glucosinolates in *A. thaliana* [10], isoflavonoid phytoalexins in *Medicago truncatula, G. echinata* and*, Lotus japonicus* [9], sesamin in *Sesamum spp.* [11]
CYP76	42	42	Biosynthesis of monoterpene volatiles in *A. thaliana* [12], monoterpene indole alkaloids in *Catharanthus roseus* [13,14], sesquiterpene volatiles in sandalwood (*Santalum album*) [15], phytoalexins in rice (*Oryza sativa*) [16,17], tanshinones in Chinese sage (*Salvia miltiorrhiza*)[18], pigment betalain in beetroot (*Beta vulgaris*) [19].Metabolism of xenobiotics in *A. thaliana* [20].
CYP72	36	33	Biosynthesis of monoterpene indole alkaloids in *C. roseus* [14,21], glycyrrhizin in licorice (*Glycyrrhiza*) [22], saponins in *M. trucantula* [23].
CYP79	26	25	Biosynthesis of cyanogenic glucosides in sorghum [24], cassava (*M. esculenta*) [25] and other plant species, glucosinolates in brassicaceae [26–28].
CYP89	25	21	Chlorophyll degradation in *A. thaliana* [29].
CYP75	24	23	Biosynthesis of flavonoids in *Petunia x hybrida*, *A. thaliana, Gentiana triflora, C. roseus, etc*. [9]
CYP716	23	12	Biosynthesis of saponins in *M. trucantula* [30] and *Maesa lanceolata* [31,32].
CYP706	21	19	Biosyntheis of sesquiterpenoids in cotton (*Gossypium arboreum*) [33].
CYP87	20	15	Biosynthesis of saponins in *Maesa lanceolata* [31].
CYP714	16	13	Degradation or biosynthesis of hormones (gibberelins) in rice (*O. sativa*) [34].
CYP736	13	11	Unknown. Pathogen response in grapevine *V. vinifera* [35].
CYP728	11	9	Unknown.
CYP80	10	9	Alkaloid biosynthesis in barberry *(Berberis stolonifera)* [36] and California poppy (*Eschscholzia californica*) [37].
CYP96	9	9	Biosynthesis of cuticular wax in *A. thaliana* [38].
CYP721	8	8	Unknown.
CYP74	7	7	Biosynthesis of jasmonates and C6 volatiles in *A. thaliana* and other plants [39–41].
CYP92	7	5	Unknown.
CYP93	7	6	Biosynthesis of flavonoids in soybean (*G. max*), *Glycyrrhiza echinata*, *Gerbera hybrid*, *Antirrhinum majus*, *Torrenia hybrid*, etc. [9,42]
CYP712	6	5	Unknown.

References specifically cited in S1 Table

1. Lee S, Badieyan S, Bevan DR, Herde M, Gatz C, Tholl D. Herbivore-induced and floral homoterpene volatiles are biosynthesized by a single P450 enzyme (CYP82G1) in Arabidopsis. Proc Natl Acad Sci U S A. 2010;107: 21205–10. doi:10.1073/pnas.1009975107
2. Beaudoin G a W, Facchini PJ. Isolation and characterization of a cDNA encoding (S)-cis-N-methylstylopine 14-hydroxylase from opium poppy, a key enzyme in sanguinarine biosynthesis. Biochem Biophys Res Commun. 2013;431: 597–603. doi:10.1016/j.bbrc.2012.12.129
3. Dang T-TT, Facchini PJ. CYP82Y1 Is N-Methylcanadine 1-Hydroxylase, a Key Noscapine Biosynthetic Enzyme in Opium Poppy. J Biol Chem. 2014;289: 2013–2026. doi:10.1074/jbc.M113.505099
4. Farrow SC, Hagel JM, Beaudoin G a W, Burns DC, Facchini PJ. Stereochemical inversion of (S)-reticuline by a cytochrome P450 fusion in opium poppy. Nat Chem Biol. Nature Publishing Group; 2015;11: 728–732. doi:10.1038/nchembio.1879
5. Haudenschild C, Schalk M, Karp F, Croteau R. Functional expression of regiospecific cytochrome P450 limonene hydroxylases from mint (Mentha spp.) in Escherichia coli and Saccharomyces cerevisiae. Arch Biochem Biophys. 2000;379: 127–36. doi:10.1006/abbi.2000.1864
6. Jørgensen K, Morant AV, Morant M, Jensen NB, Olsen CE, Kannangara R, et al. Biosynthesis of the cyanogenic glucosides linamarin and lotaustralin in cassava: isolation, biochemical characterization, and expression pattern of CYP71E7, the oxime-metabolizing cytochrome P450 enzyme. Plant Physiol. 2011;155: 282–92. doi:10.1104/pp.110.164053
7. Larbat R, Hehn A, Hans J, Schneider S, Jugdé H, Schneider B, et al. Isolation and functional characterization of CYP71AJ4 encoding for the first P450 monooxygenase of angular furanocoumarin biosynthesis. J Biol Chem. 2009;284: 4776–85. doi:10.1074/jbc.M807351200
8. Teoh KH, Polichuk DR, Reed DW, Nowak G, Covello PS. Artemisia annua L. (Asteraceae) trichome-specific cDNAs reveal CYP71AV1, a cytochrome P450 with a key role in the biosynthesis of the antimalarial sesquiterpene lactone artemisinin. FEBS Lett. 2006;580: 1411–6. doi:10.1016/j.febslet.2006.01.065
9. Ayabe S, Akashi T. Cytochrome P450s in flavonoid metabolism. Phytochem Rev. 2006;5: 271–282. doi:10.1007/s11101-006-9007-3
10. Pfalz M, Vogel H, Kroymann J. The Gene Controlling the Indole Glucosinolate Modifier1 Quantitative Trait Locus Alters Indole Glucosinolate Structures and Aphid Resistance in Arabidopsis. Plant Cell. 2009;21: 985–999. doi:10.1105/tpc.108.063115
11. Ono E, Nakai M, Fukui Y, Tomimori N, Fukuchi-Mizutani M, Saito M, et al. Formation of two methylenedioxy bridges by a Sesamum CYP81Q protein yielding a furofuran lignan, (+)-sesamin. Proc Natl Acad Sci. 2006;103: 10116–10121. doi:10.1073/pnas.0603865103
12. Boachon B, Junker RR, Miesch L, Bassard J-E, Höfer R, Caillieaudeaux R, et al. CYP76C1 (Cytochrome P450)-Mediated Linalool Metabolism and the Formation of Volatile and Soluble Linalool Oxides in Arabidopsis Flowers: A Strategy for Defense against Floral Antagonists. Plant Cell. 2015;27: 2972–90. doi:10.1105/tpc.15.00399
13. Collu G, Unver N, Peltenburg-Looman AMG, van der Heijden R, Verpoorte R, Memelink J. Geraniol 10-hydroxylase, a cytochrome P450 enzyme involved in terpenoid indole alkaloid biosynthesis. FEBS Lett. 2001;508: 215–220. doi:10.1016/S0014-5793(01)03045-9

14. Miettinen K, Dong L, Navrot N, Schneider T, Burlat V, Pollier J, et al. The seco-iridoid pathway from Catharanthus roseus. Nat Commun. 2014;5: 3606. doi:10.1038/ncomms4606

15. Diaz-Chavez ML, Moniodis J, Madilao LL, Jancsik S, Keeling CI, Barbour EL, et al. Biosynthesis of Sandalwood Oil: Santalum album CYP76F Cytochromes P450 Produce Santalols and Bergamotol. PLoS One. 2013;8: e75053. doi:10.1371/journal.pone.0075053

16. Swaminathan S, Morrone D, Wang Q, Fulton DB, Peters RJ. CYP76M7 is an ent-cassadiene C11alpha-hydroxylase defining a second multifunctional diterpenoid biosynthetic gene cluster in rice. Plant Cell. 2009;21: 3315–25. doi:10.1105/tpc.108.063677

17. Wang Q, Hillwig ML, Okada K, Yamazaki K, Wu Y, Swaminathan S, et al. Characterization of CYP76M5-8 indicates metabolic plasticity within a plant biosynthetic gene cluster. J Biol Chem. 2012;287: 6159–68. doi:10.1074/jbc.M111.305599

18. Guo J, Zhou YJ, Hillwig ML, Shen Y, Yang L, Wang Y, et al. CYP76AH1 catalyzes turnover of miltiradiene in tanshinones biosynthesis and enables heterologous production of ferruginol in yeasts. Proc Natl Acad Sci. 2013; 1–6. doi:10.1073/pnas.1218061110

19. Hatlestad GJ, Sunnadeniya RM, Akhavan N a, Gonzalez A, Goldman IL, McGrath JM, et al. The beet R locus encodes a new cytochrome P450 required for red betalain production. Nat Genet. Nature Publishing Group; 2012;44: 816–20. doi:10.1038/ng.2297

20. Hofer R, Boachon B, Renault H, Gavira C, Miesch L, Iglesias J, et al. Dual function of the cytochrome P450 CYP76 family from Arabidopsis thaliana in the metabolism of monoterpenols and phenylurea herbicides. Plant Physiol. 2014;166: 1149–1161. doi:10.1104/pp.114.244814

21. Irmler S, Schröder G, St-Pierre B, Crouch NP, Hotze M, Schmidt J, et al. Indole alkaloid biosynthesis in Catharanthus roseus: New enzyme activities and identification of cytochrome P450 CYP72A1 as secologanin synthase. Plant J. 2000;24: 797–804. doi:10.1046/j.1365-313X.2000.00922.x

22. Seki H, Sawai S, Ohyama K, Mizutani M, Ohnishi T, Sudo H, et al. Triterpene functional genomics in licorice for identification of CYP72A154 involved in the biosynthesis of glycyrrhizin. Plant Cell. 2011;23: 4112–23. doi:10.1105/tpc.110.082685

23. Biazzi E, Carelli M, Tava A, Abbruscato P, Losini I, Avato P, et al. CYP72A67 catalyses a key oxidative step in Medicago truncatula hemolytic saponin biosynthesis. Mol Plant. Elsevier Ltd; 2015;8: 1493–1506. doi:10.1016/j.molp.2015.06.003

24. Clausen M, Kannangara RM, Olsen CE, Blomstedt CK, Gleadow RM, Jørgensen K, et al. The bifurcation of the cyanogenic glucoside and glucosinolate biosynthetic pathways. Plant J. 2015;84: 558–573. doi:10.1111/tpj.13023

25. Andersen MD. Cytochromes P-450 from Cassava (Manihot esculenta Crantz) Catalyzing the First Steps in the Biosynthesis of the Cyanogenic Glucosides Linamarin and Lotaustralin. J Biol Chem. 2000;275: 1966–1975. doi:10.1074/jbc.275.3.1966

26. Mikkelsen MD, Hansen CH, Wittstock U, Halkier B a. Cytochrome P450 CYP79B2 from Arabidopsis catalyzes the conversion of tryptophan to indole-3-acetaldoxime, a precursor of indole glucosinolates and indole-3-acetic acid. J Biol Chem. 2000;275: 33712–7. doi:10.1074/jbc.M001667200

27. Wittstock U. Cytochrome P450 CYP79A2 from Arabidopsis thaliana L. Catalyzes the Conversion of L-Phenylalanine to Phenylacetaldoxime in the Biosynthesis of Benzylglucosinolate. J Biol Chem. 2000;275: 14659–14666. doi:10.1074/jbc.275.19.14659

28. Hansen CH. Cytochrome P450 CYP79F1 from Arabidopsis Catalyzes the Conversion of

Dihomomethionine and Trihomomethionine to the Corresponding Aldoximes in the Biosynthesis of Aliphatic Glucosinolates. J Biol Chem. 2001;276: 11078–11085. doi:10.1074/jbc.M010123200

29. Christ B, Sussenbacher I, Moser S, Bichsel N, Egert A, Muller T, et al. Cytochrome P450 CYP89A9 Is Involved in the Formation of Major Chlorophyll Catabolites during Leaf Senescence in Arabidopsis. Plant Cell. 2013;25: 1868–1880. doi:10.1105/tpc.113.112151

30. Carelli M, Biazzi E, Panara F, Tava A, Scaramelli L, Porceddu A, et al. Medicago truncatula CYP716A12 is a multifunctional oxidase involved in the biosynthesis of hemolytic saponins. Plant Cell. 2011;23: 3070–81. doi:10.1105/tpc.111.087312

31. Moses T, Pollier J, Faizal A, Apers S, Pieters L, Thevelein JM, et al. Unravelling the Triterpenoid Saponin Biosynthesis of the African Shrub Maesa lanceolata. Mol Plant. 2014; doi:10.1093/mp/ssu110

32. Miettinen K, Pollier J, Buyst D, Arendt P, Csuk R, Sommerwerk S, et al. The ancient CYP716 family is a major contributor to the diversification of eudicot triterpenoid biosynthesis. Nat Commun. Nature Publishing Group; 2017;8: 14153. doi:10.1038/ncomms14153

33. Luo P, Wang YH, Wang GD, Essenberg M, Chen XY. Molecular cloning and functional identification of (+)-delta-cadinene-8-hydroxylase, a cytochrome P450 mono-oxygenase (CYP706B1) of cotton sesquiterpene biosynthesis. Plant J. 2001;28: 95–104. doi:10.1046/j.1365-313X.2001.01133.x

34. Magome H, Nomura T, Hanada A, Takeda-Kamiya N, Ohnishi T, Shinma Y, et al. CYP714B1 and CYP714B2 encode gibberellin 13-oxidases that reduce gibberellin activity in rice. Proc Natl Acad Sci U S A. 2013;110: 1947–52. doi:10.1073/pnas.1215788110

35. Cheng DW, Lin H, Takahashi Y, Walker MA, Civerolo EL, Stenger DC. Transcriptional regulation of the grape cytochrome P450 monooxygenase gene CYP736B expression in response to Xylella fastidiosa infection. BMC Plant Biol. 2010;10: 135. doi:10.1186/1471-2229-10-135

36. Kraus PF, Kutchan TM. Molecular cloning and heterologous expression of a cDNA encoding berbamunine synthase, a C--O phenol-coupling cytochrome P450 from the higher plant Berberis stolonifera. Proc Natl Acad Sci U S A. 1995;92: 2071–2075. doi:10.1073/pnas.92.6.2071

37. Pauli HH, Kutchan TM. Molecular cloning and functional heterologous expression of two alleles encoding (S)-N-methylcoclaurine 3’-hydroxylase (CYP80B1), a new methyl jasmonate-inducible cytochrome P-450-dependent mono-oxygenase of benzylisoquinoline alkaloid biosynthesis. Plant J. 1998;13: 793–801.

38. Greer S, Wen M, Bird D, Wu X, Samuels L, Kunst L, et al. The cytochrome P450 enzyme CYP96A15 is the midchain alkane hydroxylase responsible for formation of secondary alcohols and ketones in stem cuticular wax of Arabidopsis. Plant Physiol. 2007;145: 653–667. doi:10.1104/pp.107.107300

39. Laudert D, Pfannschmidt U, Lottspeich F, Holländer-Czytko H, Weiler EW. Cloning, molecular and functional characterization of Arabidopsis thaliana allene oxide synthase (CYP 74), the first enzyme of the octadecanoid pathway to jasmonates. Plant Mol Biol. 1996;31: 323–335. doi:10.1007/BF00021793

40. Park JH, Halitschke R, Kim HB, Baldwin IT, Feldmann K a., Feyereisen R. A knock-out mutation in allene oxide synthase results in male sterility and defective wound signal transduction in Arabidopsis due to a block in jasmonic acid biosynthesis. Plant J. 2002;31: 1–12. doi:10.1046/j.1365-313X.2002.01328.x

41. Hughes RK, De Domenico S, Santino A. Plant cytochrome CYP74 family: Biochemical features,

endocellular localisation, activation mechanism in plant defence and improvements for industrial applications. ChemBioChem. 2009;10: 1122–1133. doi:10.1002/cbic.200800633

42. Du H, Ran F, Dong H-L, Wen J, Li J-N, Liang Z. Genome-Wide Analysis, Classification, Evolution, and Expression Analysis of the Cytochrome P450 93 Family in Land Plants. PLoS One. Public Library of Science; 2016;11: e0165020. doi:10.1371/journal.pone.0165020
