# Supplementary material for: Annotation, classification, genomic organization and expression of the Vitis vinifera CYPome
Source: PLoS One. 2018 Jun 28;13(6):e0199902. doi: 10.1371/journal.pone.0199902 (PMC6023221; doi:10.1371/journal.pone.0199902)
Supplement: S2 Table — (PDF) [file pone.0199902.s008.pdf]

S2 Table. Description of RNA-Seq experiments used for analysis of gene expression.

Genotypes	Tissue	Conditions	Number of datasets used	Reference
Carignan	Leaves	Infection with powdery mildew pathogen *Erysiphe nectator*	12	Jones et al., 2014; PRJNA254035
Pinot noir	Leaves	Infection with downy mildew pathogen *Plasmopara viticola*	10	Perazzolli et al., 2012; PRJNA168987
Touriga Nacional, *Vitis sylvestris*	Flowers	Development	23	Ramos et al., 2014; PRJNA244752
Tannat	Berries	Development	4	Da Silva et al., 2013; PRJNA203687
Shiraz	Berries	Development	4	Sweetman et al., 2012
Corvina	Berries	Development	2	Venturini et al., 2013; PRJNA169607
Riesling, Gewurztraminer	Berries	Development	4	PRJNA378596
Sangiovese, Barbera, Negro amaro, Refosco	Berries	Development	48	Palumbo et al., 2014; PRJNA265040
